# Biomolecule–polymer conjugates synthesized by a combination of organometallic bioconjugation and atom transfer radical polymerization

**DOI:** 10.1039/d6py00714g

**Published:** 2026-10-05

**Authors:** Hayden R. Montgomery, Tong Zhang, Grace E. Kunkel, Magdalena F. Polite, Jeonghun Lee, Marco S. Messina, Alexander M. Spokoyny, Heather D. Maynard

**Affiliations:** a Department of Chemistry and Biochemistry, University of California, Los Angeles 607 Charles E. Young Drive East Los Angeles California 90095-1569 USA hmaynard@ucla.edu; b Department of Chemistry and Biochemistry, University of Delaware, 102 Brown Laboratory Newark DE 19716 USA; c California NanoSystems Institute, University of California, Los Angeles 570 Westwood Plaza Los Angeles California 90095-1569 USA; d Department of Bioengineering, University of California, Los Angeles 420 Westwood Plaza Los Angeles California 90095-1600 USA

## Abstract

Organometallic Au(iii) cysteine arylation strategies have recently been employed for efficient and selective conjugation to proteins and peptides. In this work, atom transfer radical polymerization (ATRP) was utilized to synthesize iodophenyl end-functionalized poly(polyethylene glycol methacrylate) (pPEGMA), poly(hydroxyethyl methacrylate) (pHEMA) and poly(2-(dimethylamino)ethyl methacrylate) (pDMAEMA), which were subjected to MeDalPhos–Au(i)Cl to form Au(iii) polymer reagents. The Au(iii)–pPEGMA was reacted with T4-lysozyme (T4L) containing a free thiol to form a T4L–pPEGMA conjugate. In a complementary approach, an Au(iii) ATRP initiator was synthesized for subsequent conjugation to a peptide and T4L to form biomacroinitators. These peptide and protein macroinitiators were then subjected to polymerization conditions to form pDMAEMA conjugates *in situ*. This work demonstrates that Au(iii) organometallic reagents in conjunction with ATRP offers an efficient strategy for the preparation of protein–polymer conjugates by either grafting to or grafting from approaches.

## Abstract 2

This study demonstrates that oxidative addition with gold complexes, combined with ATRP, enables protein–polymer conjugate synthesis by grafting to and grafting from, offering an additional method for advancing biomaterial synthesis.

## Graphical abstract

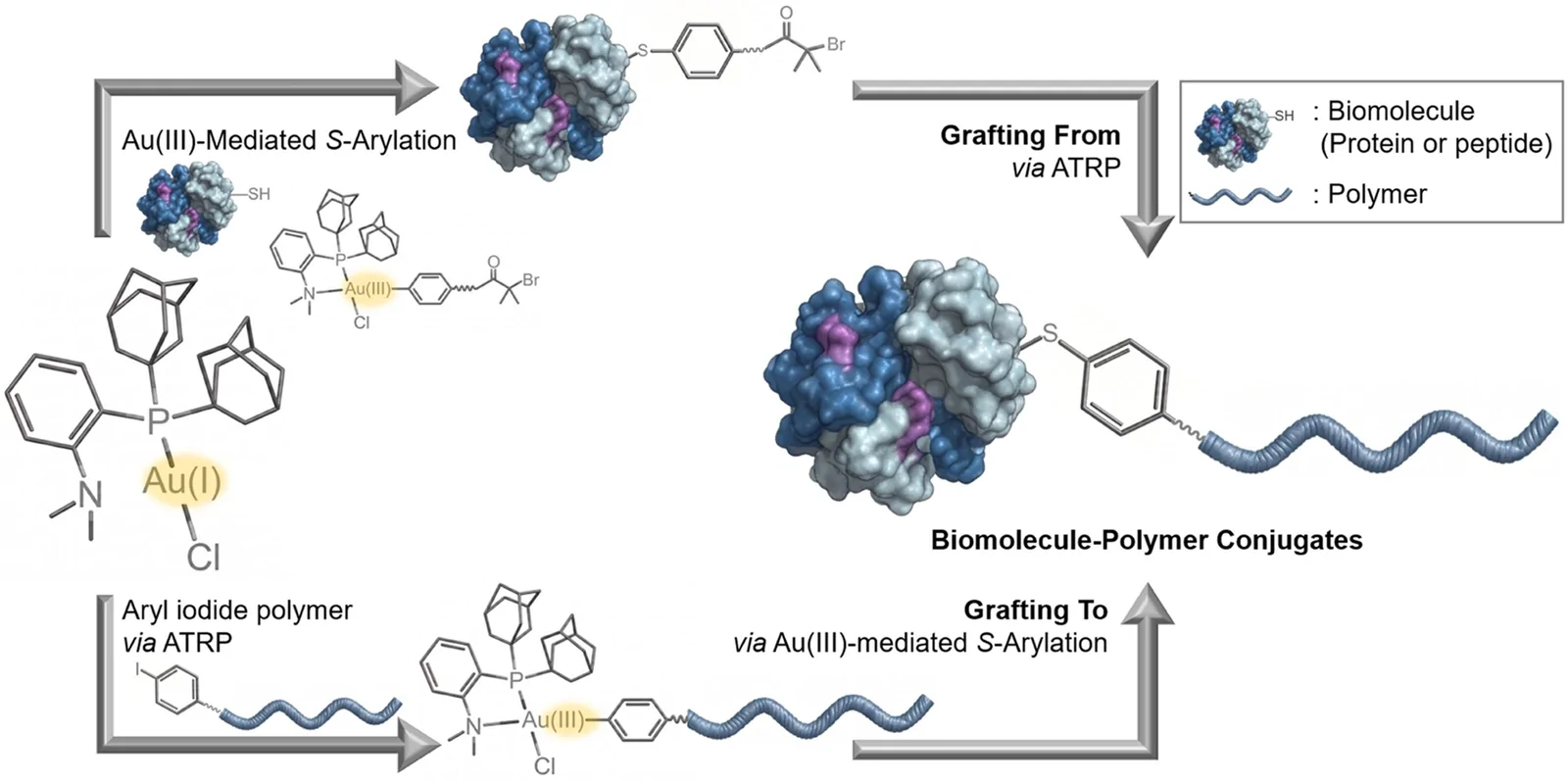

## Introduction

Biomolecule–polymer conjugates represent an important class of therapeutics, with many PEGylated proteins and peptides being used in the clinic to treat diseases.^1^ While PEGylated conjugates are the only Federal Drug Administration (FDA) approved biomolecule–polymer conjugate drugs to date, much work has been undertaken to study the effect of alternative polymers on biomolecule materials properties.^2–4^ Additionally, the pharmacokinetic properties of various polymer backbones and side chain types have been widely studied, and have potential to improve therapeutic properties of protein drugs.^2,3^ Protein–polymer conjugate therapeutics benefit from the use of polymers with well-defined molecular weight distributions as they can improve reproducibility and safety. To this end, controlled polymerization techniques have been developed to provide polymers with predictable molecular weights and narrow dispersities.^5^ Atom transfer radical polymerization (ATRP) is one such technique that has been widely exploited to make protein–polymer conjugates *via* grafting to and grafting from methods.^6,7^ In the former, the end-functionalized polymer is synthesized first and conjugated to the protein, and in the latter, the initiator is placed on the protein and the polymer is formed *in situ*.^1,8–11^

Historically, protein conjugation strategies have evolved alongside small-molecule biorthogonal synthetic techniques.^12,13^ Organometallic biomolecular conjugation strategies have become invaluable tools for producing modified proteins in an efficient manner. These methods use stochiometric or catalytic metal-based complexes to covalently attach polymers or small molecules to proteins, offering precise control under mild conditions, while preserving protein function. Typically, bioconjugations target nucleophilic amino acid residues such as lysine (Lys), cysteine (Cys), tyrosine (Tyr), and histidine (His), as well as the N-terminus of proteins and peptides.^12,14,15^ Building on the success of the Cu-catalyzed azide–alkyne cycloaddition (CuAAC),^16–18^ further advancements in organometallic bioconjugation have been explored for C–H arylation,^19,20^ C–C,^21^ and C–S^22,23^ cross-coupling. Recently, gold-mediated organometallic approaches have gained significant interest in bioconjugation due to their fast reactivity, exceptional selectivity, compatibility with aqueous systems, and mild reaction conditions. Several groups demonstrated this approach in peptide and protein bioconjugation utilizing chemoselective cysteine modification *via* ligand-controlled C–S bond-forming reductive elimination using cyclometallated Au(iii) systems.^22,24–26^ Recently, we and others have developed organometallic gold-complexes using bulky hemilabile bidentate P,N ligands as highly selective reagents for forming stable bioconjugates with extremely fast reaction kinetics.^27–35^

Au(iii) organometallic oxidative addition (OA) complexes in particular have shown excellent chemoselectivity for Cys residues, resulting in the efficient transfer of aryl groups to peptides and proteins.^29,31,32,34^ We demonstrated that these OA complexes could be used to attach oligomers or polymers to biomolecules.^27,29,34^ For example, Au(iii)-modified mono-methoxy-polyethylene glycol (mPEG) was coupled to Cys containing proteins with near equimolar amounts of reagent, in one minute, at room temperature, and at low µM concentrations (Scheme 1a).^34^ The Au(iii)–mPEG reagents were synthesized from the corresponding mPEG polymers with aryl iodide end groups, which were synthetically installed post-polymerization. While highly successful for PEGylation, exploring other polymers synthesized *via* controlled radical polymerizations would further expand the versatility of Au(iii)-mediated bioconjugation. In this study, the chemocompatibility and efficiency of the Au(iii) *S*-arylation process was explored in the context of polymers synthesized by ATRP (Scheme 1b), which are more chemically and materially complex than PEG.^36^

**Scheme 1:**
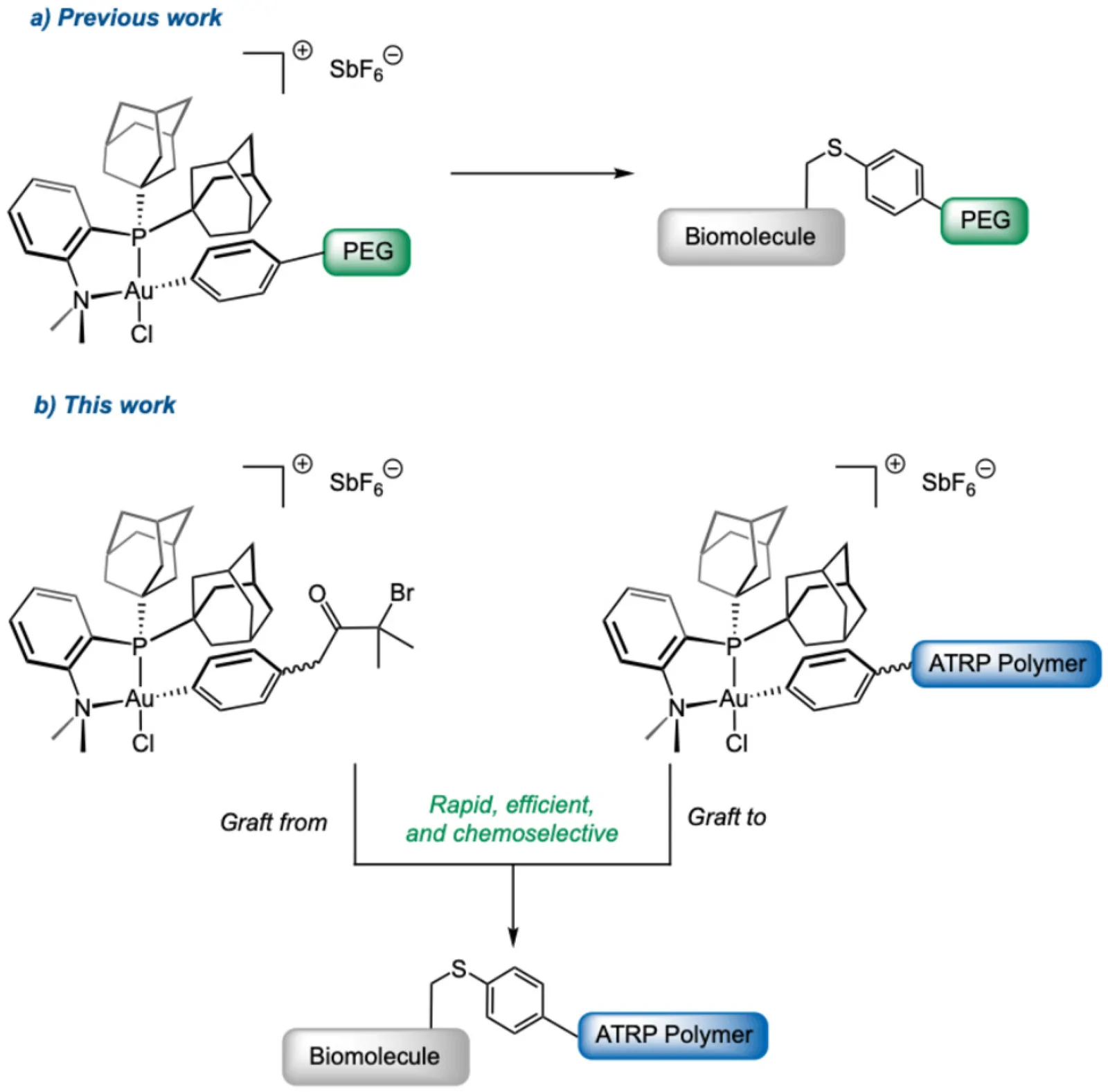
(a) Schematic of Au(iii)-mediated cysteine PEGylation^32^ (b) This work representing beyond PEGylation use of Au(iii)–ATRP initiators for graft to and graft from conjugation approaches.

## Results and discussion

### Preparation of aryl-iodide-containing ATRP initiator

An ATRP initiator containing an aryl-iodide end group was synthesized to prepare aryl-iodide terminated polymers that could be subsequently reacted to form Au(iii) complexes (Fig. 1a). First, 4-iodophenol was coupled to *N*-Boc-2-bromoethylamine, followed by subsequent deprotection to afford the free amine as a TFA salt adduct 1. The aryl-iodide amine was then coupled to α-bromoisobutyryl bromide to afford the ATRP initiator 2. This initiator was then used directly to make polymers for the grafting to approach. It should be noted that oxidative addition of the Au(i) complex to the aryl iodide initiator 2 prior to ATRP was attempted, but the polymerization was unsuccessful (data not shown). Additionally, to expand the scope of applications, the benzylamine-linked initiator 2′ was synthesized in a single step for subsequent bioconjugation (Fig. 1b).

**Fig. 1:**
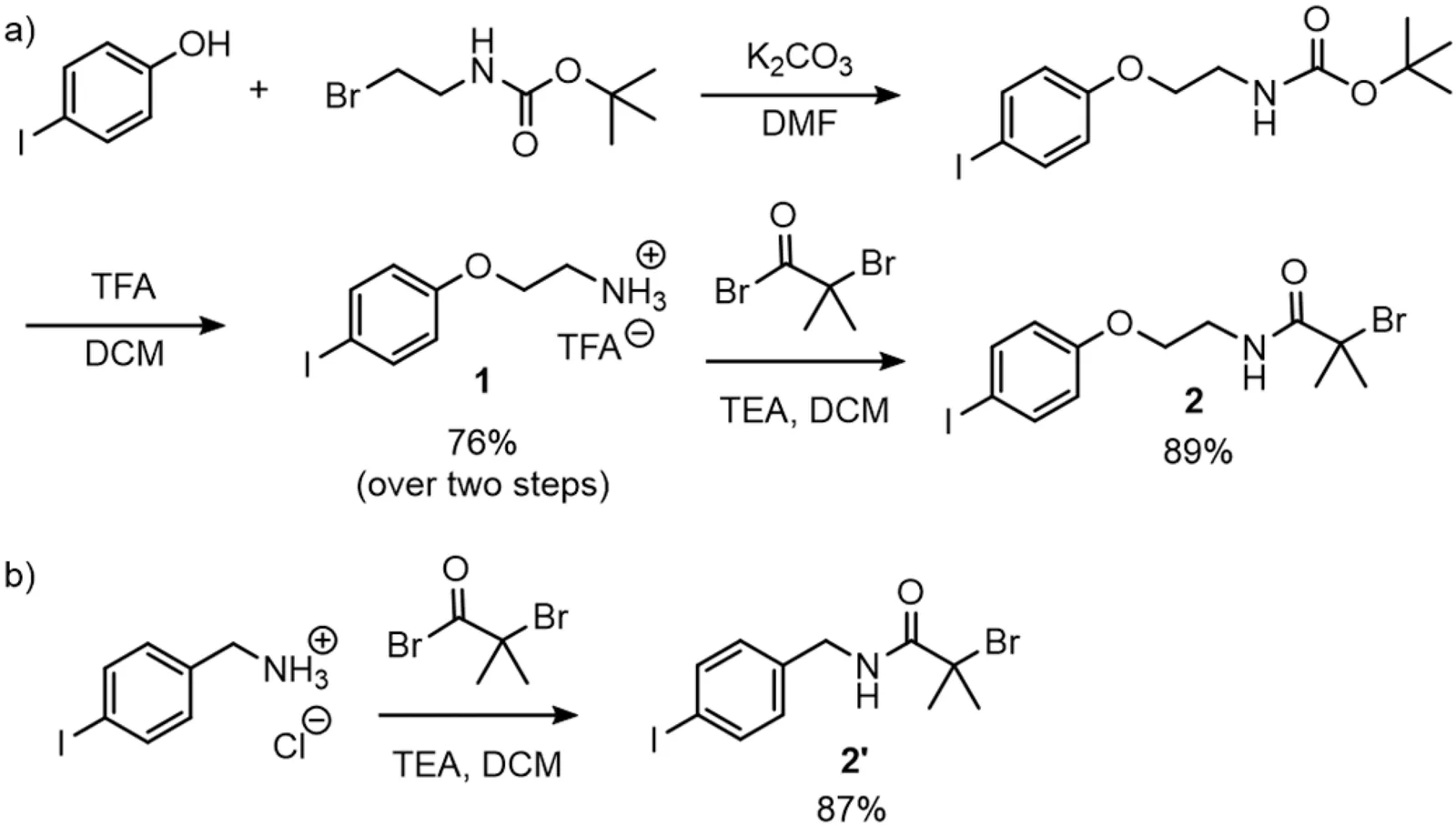
Synthesis of aryl iodide-functionalized ATRP initiators: (a) preparation of amide-linked initiator 2*via* a three-step route involving etherification and deprotection, and (b) preparation of benzylamide-linked initiator 2′. DMF = dimethylformamide, TFA = trifluoroacetic acid, DCM = dichloromethane, TEA = triethylamine.

### Preparation of aryl-iodide terminated polymers

We next deployed initiator 2 to synthesize an array of aryl-iodide terminated polymers based on either polyethylene glycol methacrylate (PEGMA), 2-(dimethylamino)ethyl methacrylate (DMAEMA), or hydroxyethyl methacrylate (HEMA) monomers *via* typical ATRP conditions.^34^ Specifically, PEGMA, HEMA or DMAEMA were combined with Cu(i)Cl and 2,2-bypyridine in methanol followed by the addition of the aryl iodide initiator to form the polymers (Fig. 2a). Number average molecular weights (*M*_n_) of 29.5, 7.5, and 48.4 kDa with dispersities (*Đ*) of 1.33, 1.10, and 1.63 were measured for pPEGMA, pDMAEMA, and pHEMA, respectively, *via* SEC using PMMA standards and DMF as the eluent (Table 1 entries 1, 3 and 4, SEC traces Fig. 2b, S11, S13, molecular weights by ^1^H NMR also provided on the table). Complete retention of the aryl-iodide end group was observed in all cases *via*^1^H NMR (Fig. S8–S10 and S12). To further demonstrate the versatility and catalytic tolerance of this platform, the benzylamine-linked initiator 2′ was also successfully employed. For instance, the ATRP of PEGMA using 2′ proceeded even under an alternative CuCl/TPMA catalytic system, yielding pPEGMA with a number-average molecular weight (*M*_n_) of 25.3 kDa and a dispersity (*Đ*) of 1.37 (Table 1 entry 2). Crucially, the polymerization remained highly consistent with the results obtained from initiator 2, confirming that the aryl-iodide initiating systems are robust and adaptable across various ATRP formulations. This polymer was further characterized as shown in Fig. S9.

**Fig. 2:**
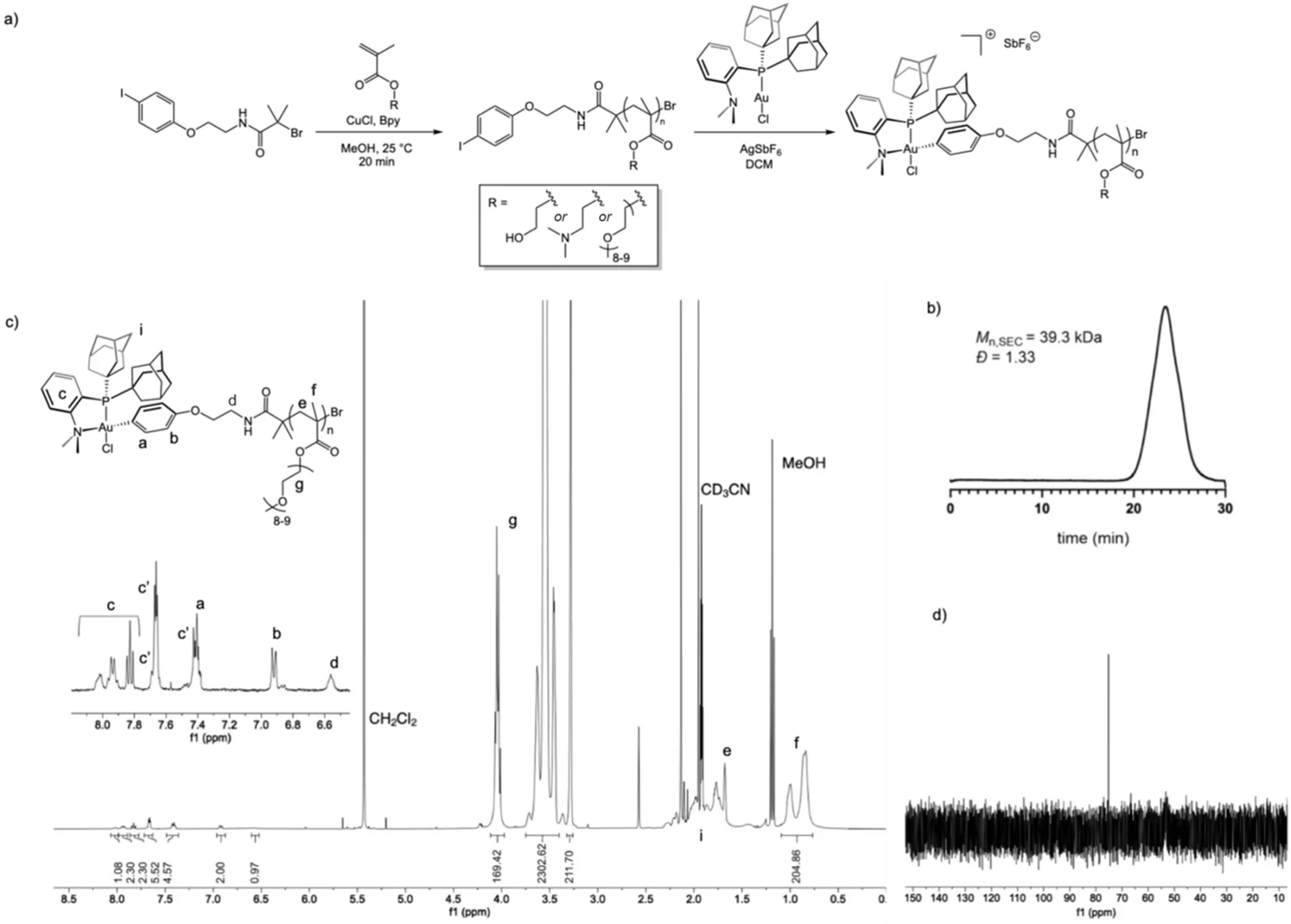
(a) Synthetic scheme of ATRP conditions for the synthesis of pPEGMA, pDMAEMA, and pHEMA and subsequent oxidative addition to form the corresponding Au(iii) polymer reagents. (b) SEC of pPEGMA aryl iodide polymer indicating a molecular weight of 29.5 kDa and a dispersity of 1.33. (c) ^1^H NMR of Au(iii)–pPEGMA polymer. (d) ^31^P{^1^H}NMR of Au(iii)–pPEGMA showing conversion to the Au(iii)–polymer reagent. Note: In (c), peaks c′ represent HAr in unreacted MeDalPhos–Au(i)Cl.

**Table 1:** Polymer data for grafting to and grafting from^a^

Entry	Monomer	Initiator	*M*_n,NMR_ kDa	*M*_n,SEC_ kDa	*Đ*
1	PEGMA	2	31.0	29.5	1.33
2	PEGMA	2′	25.3	27.8	1.37
3	DMAEMA	2	10.9	7.5	1.10
4	HEMA	2	29.2	48.4	1.63
5	DMAEMA	4	6.1	253	1.61
6	DMAEMA	5	—	62.2	1.79

a Entries 1–6, polymers were analyzed by SEC (DMF as eluent) calibrated using PMMA standards. Entry 6, the polymer was obtained after proteinase K digestion.

### Synthesis of ATRP polymers with a Au(iii) end-group

Purified aryl-iodide terminated polymers were then treated with MeDalPhos–Au(i)Cl and AgSbF_6_ in dichloromethane (DCM) for 14 hours at room temperature to generate the corresponding Au(iii)–polymer reagents (Fig. 2a). Notably, poor solubility of the pHEMA polymer hindered conversion to the Au(iii) product. The addition of polar co-solvents was explored to assist in solubilizing pHEMA; however, a significant decrease in conversion along with concomitant gold nanoparticle formation was observed when adding >10% MeOH, MeCN, or acetone. For this reason, OA was exclusively performed in 100% DCM for both pPEGMA and pDMAEMA after formation of the polymer. Though pDMAEMA was soluble in DCM, introduction of AgSbF_6_ did not result in the expected precipitation of AgCl, even after 14 hours, and OA did not proceed to a significant extent as observed by ^31^P NMR (data not shown). The precipitation of AgCl is generally an indication that the chloride has been abstracted from the Au(i) center, and the precipitation process drives OA forward. We hypothesized that the lack of product conversion was due to the interaction of Ag ions with the pendant dimethylamine monomer units, resulting in the incompatibility of this side chain type with the OA process. While pPEGMA polymers have branched structures leading to more complex materials properties than linear mPEG polymers, the two have very similar chemical functionality and linear mPEG was previously reported to perform well in the Au(i) to Au(iii) OA process. Similarly, the OA of the pPEGMA polymers was successfully undertaken with slight excess of Au(i), and the pPEGMA–Au(iii) reagent was isolated without purification from excess Au(i) and stored in air for subsequent bioconjugation reactions (^1^H NMR Fig. 2c, ^31^P NMR spectroscopy showing the signal corresponding to the product Fig. 2d). Complete characterization of the pPEGMA Au(iii)-reagent synthesized from initiator 2′ is also provided in Fig. S14.

### Grafting to conjugation of Au(iii) polymers to biomolecules

The pPEGMA–Au(iii) reagent was then conjugated to the model protein T4 lysozyme (T4L) V131C mutant, which contains a single Cys residue. Treatment of T4L with pPEGMA–Au(iii) (1.3 equiv.) at 76 µM T4L concentration in DPBS at 25 °C resulted in the formation of the T4L–pPEGMA conjugate as determined by SDS-PAGE (Fig. 3a). A distinct band corresponding to the conjugate was visualized using both iodine and Coomassie staining, confirming the presence of pPEGMA and the protein–polymer conjugate, respectively, (Fig. 3b) demonstrating that the polymers were covalently attached to the protein *via* OA with 93% conversion (see Fig. S15 for calculation). For the pPEGMA–Au(iii) prepared *via* initiator 2′, the T4L conjugation was conducted using the same protocol and a nearly identical conversion of 92% was achieved (see Fig. S16 for SDS PAGE results).

**Fig. 3:**
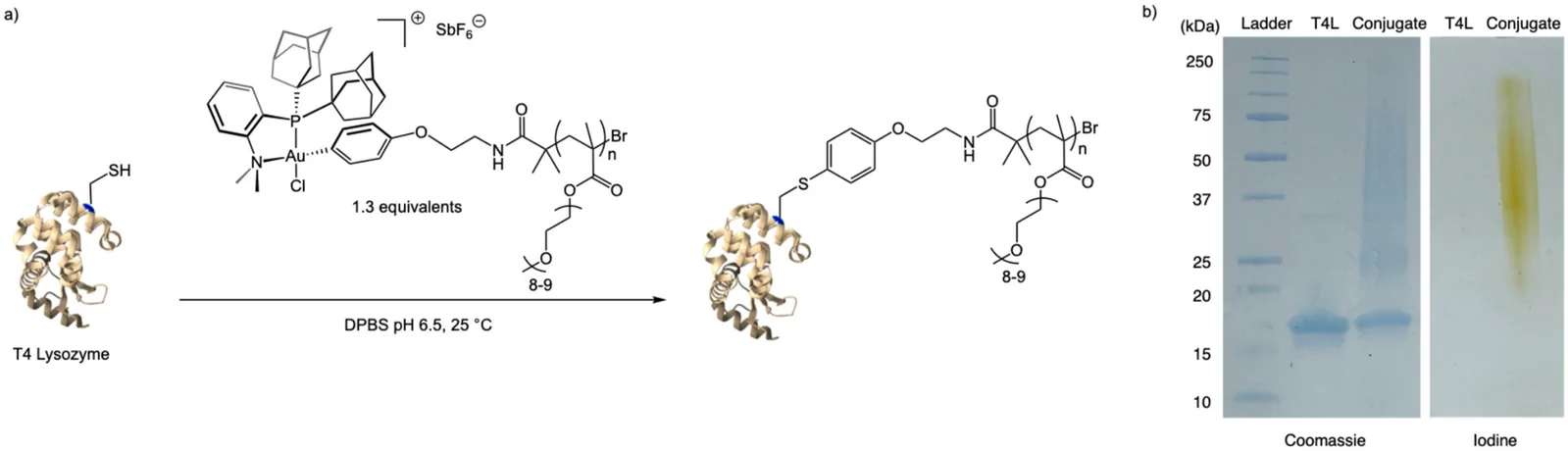
(a) Scheme of cysteine arylation grafting to bioconjugation of T4 lysozyme and pPEGMA Au(iii)-reagent. (b) SDS-PAGE of T4L at ∼18 kDa and corresponding conjugate indicated by a smeared band at larger molecular weight (left Coomasie staining, right iodine staining). Note: Protein structures were generated from PDB ID 2huk,^37^ created with ChimeraX.^38^

### Synthesis of Au(iii)–ATRP initiator

Since the grafting to approach was not successful for pDMAEMA, we sought to create pDMAEMA–biomolecule conjugates *via* the grafting from strategy. pHEMA was not explored further due to its insolubility in aqueous solution, which would confound results. An Au(iii) ATRP initiator 3 was synthesized from the corresponding aryl-iodide initiator 2. In general, organometallic OA processes have been shown to undergo insertion across both alkyl and aryl C–X bonds. We hypothesized that the chemoselectivity of Au(i) reagents would enable selective OA across the aryl-iodide bond leaving the tertiary alkyl bromide group present in 3 unperturbed and allowing for subsequent polymerization. Treatment of 2 with MeDalPhos–Au(i)Cl and AgSbF_6_ in DCM for 15 minutes resulted in complete conversion of MeDalPhos–Au(i)Cl as monitored *via*^31^P NMR spectroscopy, to provide the ATRP initiator Au(iii) OA complex (3, Fig. 4a). This reagent was thoroughly characterized by ^1^H NMR and ^31^P{^1^H} NMR spectroscopy and electrospray ionization-mass spectrometry (ESI-MS) confirming that the tertiary bromide functionality necessary for initiation of polymerization remained fully intact throughout the OA process (Fig. 4b and c, Fig. S6, S7).

**Fig. 4:**
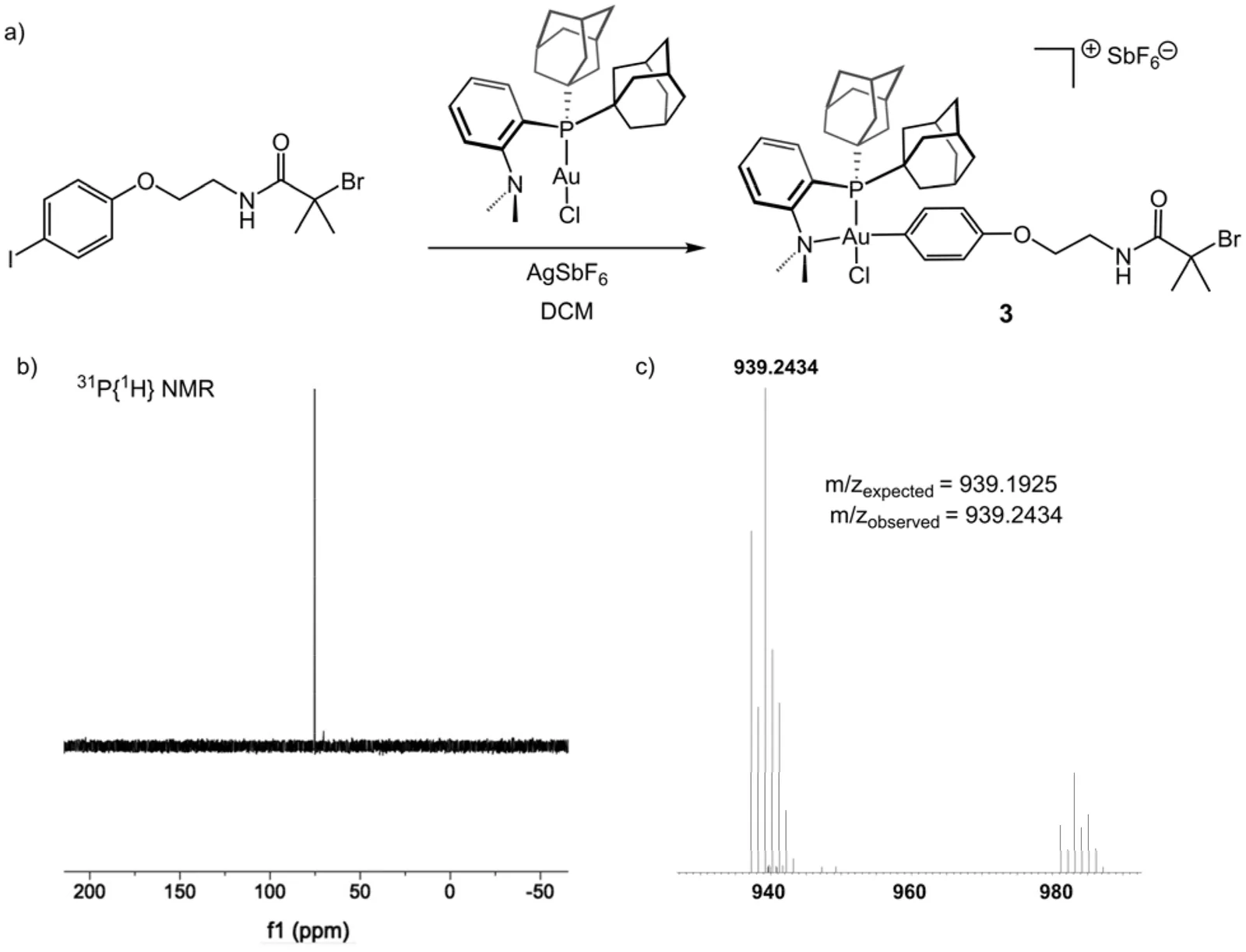
(a) Synthetic scheme of oxidative addition of ATRP initiator to prepare Au(iii) initiator. (b) ^31^P{^1^H}NMR of the Au(iii) initiator indicating complete conversion to the desired product. (c) ESI-MS of the Au(iii) complex indicating full retention of the tertiary bromide functionality.

### Synthesis of biomolecule–ATRP macroinitiators

In the grafting from approach, small-molecule initiators are covalently attached to amino acid residues on biomolecules and purified thereby creating biomolecule-initiator hybrids termed macroinitiators. Macroinitiators enable controlled polymerization processes to be carried out directly from the biomolecule surface, thereby allowing access to biomolecule–polymer conjugates that can be easily separated from small-molecule reaction components. To this end, initiator 3 was utilized for bioconjugation to the free Cys on both a peptide and a protein (Fig. 5). Limited excess Au(iii) reagent was used in order to simplify purification of the conjugate from the biomolecule–ATRP macroinitiator. Specifically, a solution of 1 mM peptide or 76 µM T4L each containing a single free Cys, was subjected to 1.2 and 1.3 equivalents of Au(iii)–ATRP initiator 3, respectively, and quantitative conversion was observed after five minutes by intact LC-MS analysis. The Au(iii) initiator was first dissolved in acetonitrile and then added to the aqueous buffer solutions in order to facilitate solubility of the gold complex in the reaction solution. This peptide macroinitiator was purified using preparative high-performance liquid chromatography (HPLC, peptide) and the protein macroinitiator by fast protein liquid chromatography (FPLC, protein) to remove the excess Au(iii) reagent, as well as Au(i) byproducts from the reaction mixtures. Pure peptide macroinitiator 4 (Fig. 5a) and T4L macroinitiator 5 (Fig. 5b) were isolated and observed *via* LC-MS. In all cases, the tertiary bromide functionality remained intact after the bioconjugation process as observed by LC-MS, indicating that this Au(iii)-mediated bioconjugation process can be used to synthesize biomolecule–ATRP macroinitiators efficiently and with high fidelity of reactive end groups.

**Fig. 5:**
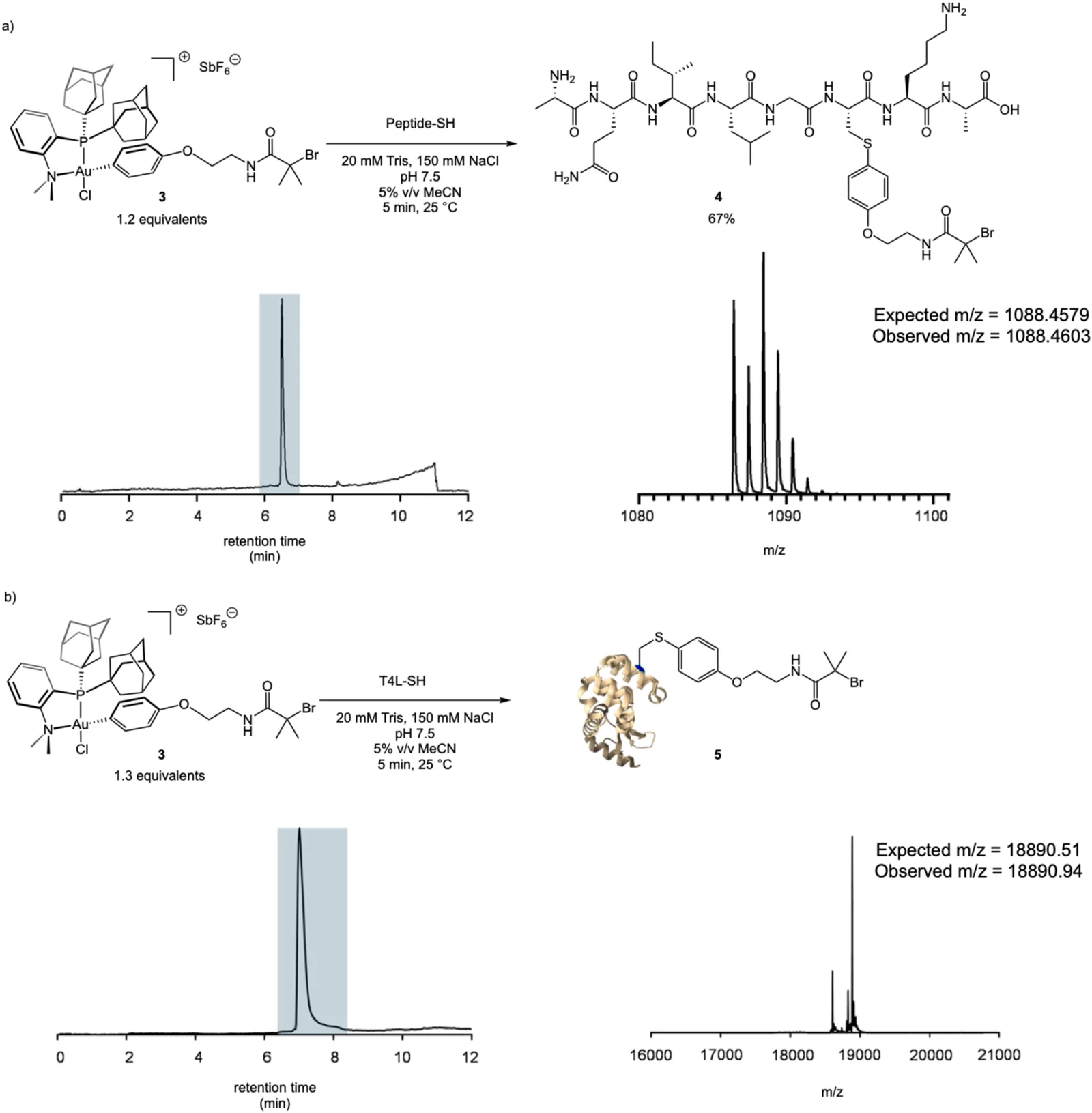
(a) Synthetic scheme of bioconjugation of the Au(iii) initiator 3 with peptide Cys. LCMS analysis demonstrates conversion to peptide macroinitiator product 4. (B) Synthetic scheme of bioconjugation of the Au(iii) initiator 3 with T4-lysozyme Cys. LCMS shows conversion to protein macroinitiator product 5. LC-MS analyses were performed using purified biomolecular macroinitiators.

### Grafting from polymerization using biomolecule–ATRP macroinitiators

Previously reported grafting from conditions using single-electron transfer-living radical polymerization (SET-LRP) were employed to synthesize pDMAEMA conjugates.^39^ Specifically, peptide macroinitiator and DMAEMA monomer were dissolved in degassed water, and added to a solution of premixed Cu(i)Br and Me_6_TREN in degassed water and the mixture was reacted at 0 °C for 20 minutes (Fig. 6a and b). The resulting conjugate was precipitated in cold diethyl ether, followed by analysis by ^1^H NMR and SEC (Fig. 6c and Fig. S21). A very large molecular weight of 253 kDa was observed by SEC with a moderate dispersity of 1.61. We suspected that the apparent high molecular weight was due to self-assembly of the peptide portion in the DMF solvent used for the SEC, providing an artificial increase in molecular weight due to comparison to PMMA standards. These hypotheses were supported by ^1^H NMR, which gave a more reasonable *M*_n_ of 6.1 kDa (Table 1, entry 5). Following successful polymerization from 4, macroinitiator 5 was then explored utilizing identical grafting from SET-LRP conditions. However, monomer conversion was unexpectedly low (data not shown), likely due to the low concentration of macroinitiator. To address this limitation, activator generated by electron transfer (AGET) ATRP was conducted in the presence of sacrificial initiator 2 to facilitate polymer growth and improve conversion efficiency, and the resulting conjugate was analyzed *via* SEC after purification by dialysis. A new peak appeared at an earlier retention time relative to the peak of unreacted 5 (Fig. S22), indicative of successful formation of the T4L–pDMAEMA conjugate. These results were further confirmed by gel electrophoresis (Fig. 6e) as well as a smear for a T4L–pDMAEMA conjugate was observed at the high molecular weight region with the presence of unreacted T4 at the low molecular weight region (lane 4). The conjugate was detected as a large smear, which is typical for protein–polymer conjugates. To confirm polymer formation, the conjugate was treated with proteinase K to cleave the protein from the T4L–pDMAEMA. SDS-PAGE showed the absence of protein bands (lane 5), suggests the successful digestion of T4L from the polymer segment. After the polymerization reaction and subsequent successful digestion of T4L with proteinase K, SEC confirmed that the polymer was synthesized from the T4L initiator (Fig. 6d). The number-average molecular weight of cleaved pDMAEMA by SEC was 62.2 kDa with a relatively high dispersity of 1.79 (Table 1, entry 6). Aqueous SEC also showed similar high dispersity (Fig. S23), while the grafting to approach gave a narrow dispersity of 1.10 (Table 1, entry 3). These results suggest that polymerization from the protein can result in broadened molecular weight distribution. In the future, the addition of salts may be possible to optimize the grafting from polymerization.^40^

**Fig. 6:**
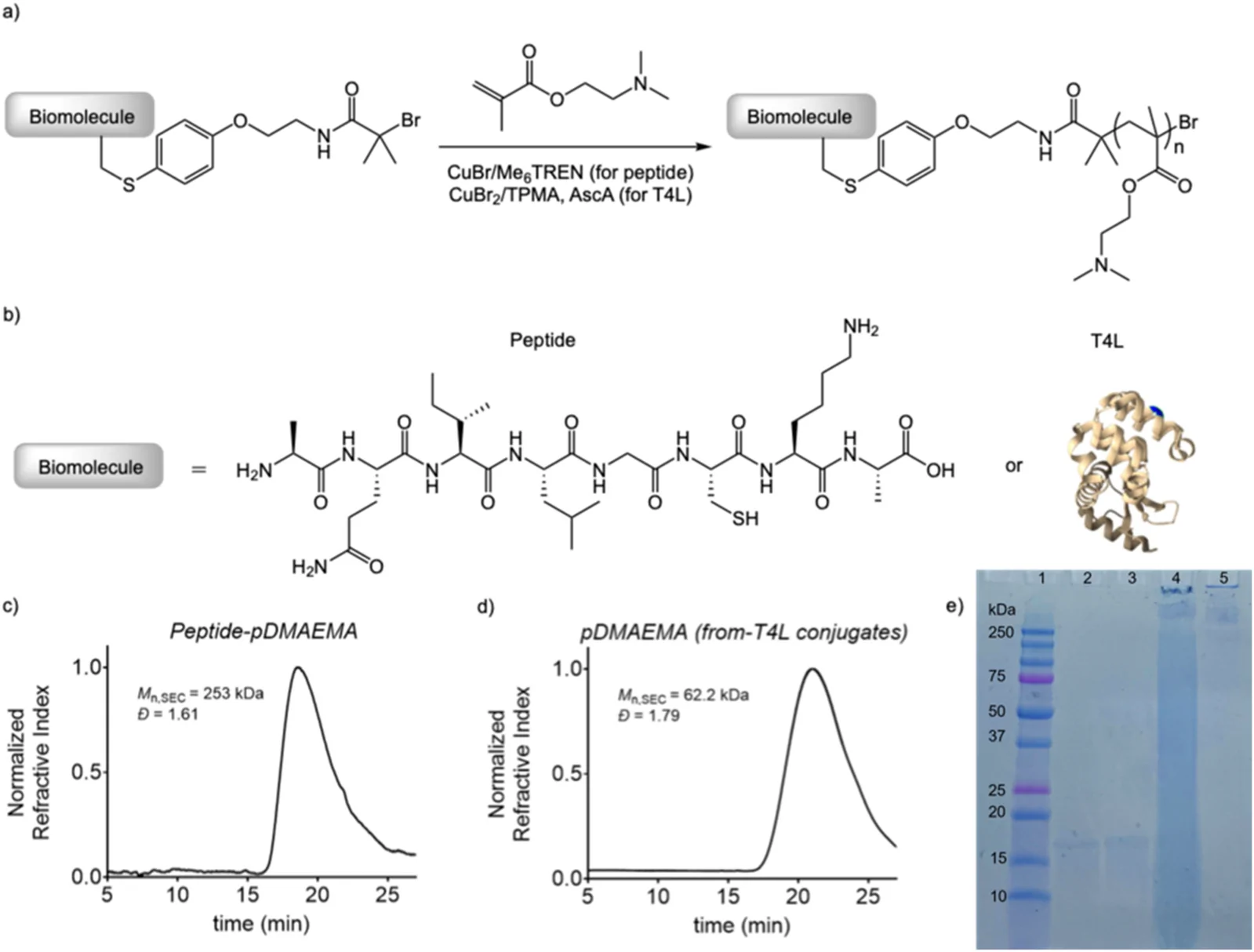
(a) Synthetic scheme of grafting from SET-LRP using biomolecule as macroinitiator. (b) Biomolecules used in the reaction. (c) SEC confirms synthesis of peptide–pDMAEMA conjugate, acquired with DMF SEC compared to PMMA standards. (d) SEC confirms synthesis of pDMAEMA (digested from T4L–pDMAEMA conjugates using proteinase K) acquired from DMF SEC compared to PMMA standards. (e) Gel electrophoresis analysis of the synthesis of T4L–pDMAEMA conjugates. Lane 1: ladder; lane 2: T4L; lane 3: T4L–ATRP initiator; lane 4: T4L–pDMAEMA; lane 5: pDMAEMA (digested form T4L–pDMAEMA conjugates using proteinase K).

In summary, we demonstrate the chemocompatibility of the Au(iii) *S*-arylation approach in the context of biomolecule polymer conjugates synthesized *via* a controlled radical polymerization technique, namely ATRP. A grafting to approach can be utilized when the chemical functionality of the polymer of choice is compatible with the OA process during the synthesis of the Au(iii)–polymer reagents; for example, we found that pPEGMA–Au(iii) was successfully synthesized. However, it is hypothesized that the OA is not amenable to polymers containing amine sidechains or polymers which are not soluble in DCM; such as pDMAEMA and pHEMA, respectively. Further investigation of the OA process or ligands on the gold complex could improve chemocompatiblity and solvent tolerance during OA, which would allow for greater accessibility to different polymer chemistries for grafting to. In contrast, the grafting from approach offers a robust and complementary alternative for the synthesis of biomolecule–polymer conjugates, including polymers containing amine side chains. To this end, both peptide and protein macroinitiators were synthesized rapidly and efficiently, with full fidelity of the tertiary halide initiation functionality. In this case, T4L–pDMAEMA, which was not possible by the grafting to strategy, was synthesized using the grafting from technique. Collectively, these results demonstrate the synthesis of different conjugates by a combination of OA with MeDalPhos–Au(i)Cl and ATRP, but that the approach (grafting to or grafting from) should be carefully considered prior to conjugation by evaluating the solubility and chemical structure of the polymer of interest.

## Conclusions

This work serves as an exploration into the synthesis of biomolecule–polymer conjugates with more complex chemical functionality and steric profiles than PEG, utilizing an Au(iii) *S*-arylation approach. The substantial flexibility afforded by the rapid kinetics and chemoselectivity of the metal-mediated bioconjugation process facilitated the creation of conjugates *via* either grafting to or grafting from, which was critical for addressing synthetic limitations. Specifically, while grafting to enabled direct conjugation of well-defined and monodisperse polymers, inefficient conjugation was observed when using amine containing polymers (pDMAEMA). This limitation was overcome through the use of grafting from, thus expanding the scope of polymer functionality enabled by our metal-mediated bioconjugation strategy. However, grafting from resulted in bioconjugates with high dispersities, thus presaging future work optimizing polymerization conditions. The synthetic ease with which a range of high-fidelity aryl-iodide and Au(iii)–ATRP initiators can be synthesized encourages further exploration of both the grafting to and grafting from approaches toward biomolecule–polymer conjugates utilizing this Au(iii)-bioconjugation approach.

## Author contributions

HRM and GEK designed experiments, HRM, TZ, GEK, MFP and JL carried out the reactions and initial data analysis. MSM did initial exploration of the approach. HDM conceived of the work. AMS and HDM oversaw the project and helped with analysis. HRM wrote the initial draft and all authors reviewed and edited.

## Conflicts of interest

There are no conflicts to declare.

## Supplementary Material

PY-017-D6PY00714G-s001

## Data Availability

All data is included in the supplementary information (SI) or can be requested from the authors. Supplementary information is available including synthesis of small molecules, polymers, and conjugates, characterization not in the main paper, and protein expression method. See DOI: https://doi.org/10.1039/d6py00714g.
