# Supplementary material for: Biomolecule–polymer conjugates synthesized by a combination of organometallic bioconjugation and atom transfer radical polymerization
Source: Polym Chem. 2026 Oct 5;17(41):4474–82. doi: 10.1039/d6py00714g (PMC13637460; doi:10.1039/d6py00714g)
Supplement: Supplementary file 1 [file PY-017-D6PY00714G-s001.pdf]

Supplementary Information (SI) for Polymer Chemistry.
This journal is © The Royal Society of Chemistry

# Supporting information

# Biomolecule-Polymer Conjugates Synthesized by a Combination of Organometallic Bioconjugation and Atom Transfer Radical Polymerization

Hayden R. Montgomery,[a] Tong Zhang,[a,c] Grace E. Kunkel,[a] Magdalena F. Polite,[a] Jeonghun Lee,[a] Marco S. Messina,[b] Alexander M. Spokoyny,[*a,c] and Heather D. Maynard [*a,c,d]

[a] Department of Chemistry and Biochemistry, University of California, Los Angeles, 607 Charles E. Young Drive East, Los Angeles, California 90095-1569, United States.

[b] Department of Chemistry and Biochemistry, University of Delaware, 102 Brown Laboratory, Newark, DE 19716, United States.

[c] California NanoSystems Institute, University of California, Los Angeles, 570 Westwood Plaza, Los Angeles, California 90095-1569, United States.

[d]Department of Bioengineering, University of California, Los Angeles, 420 Westwood Plaza, Los Angeles, California 90095-1600, United States.

*Correspondence to: maynard@chem.ucla.edu and spokoyny@chem.ucla.edu

# Table of Contents

Materials ........ 2
Instrumentation ........ 3
Synthesis of an Aryl Iodide ATRP Initiator Precursor (1) ........ 5
Synthesis of an Aryl Iodide ATRP Initiator (2) ........ 8
Synthesis of an Aryl Iodide ATRP Initiator (2') ........ 11
Synthesis of an Aryl Iodide ATRP Initiator (3) ........ 13
Synthesis of pPEGMA-ArI ........ 17
Synthesis of pHEMA-ArI ........ 20
Synthesis of pDMAEMA-ArI ........ 22
Synthesis of pPEGMA Au(III) reagent ........ 24
Solid-Phase Peptide Synthesis ........ 28
T4 Lysozyme Expression ........ 30
Au(III)-pPEGMA T4 Lysozyme Conjugation ........ 33
Peptide Macroinitiator Synthesis ........ 33
T4L Macroinitiator Synthesis ........ 33
Peptide-Polymer Conjugates via Grafting From SET-LRP ........ 34
T4L-Polymer Conjugate *via* Grafting From SET-LRP ........ 35
References ........ 38

## Materials

Unless otherwise stated, all materials were purchased and used as received from Fisher Scientific, Combi-Blocks, Alfa Aesar, Oakwood Chemicals, or Sigma Aldrich. Inhibitors were removed from the monomers by passing through an aluminum column prior to usage. Milli-Q water with a resistivity of >18.2 MΩ cm was obtained from an in-line Millipore RiOs/Origin water purification system.

## Instrumentation

Nuclear Magnetic Resonance (NMR)

NMR spectra were recorded on the following: AV400 Bruker spectrometer at 400 ($^{1}H$) and 121 MHz ($^{31}P\{^{1}H\}$); AV300 Bruker spectrometer at 300 ($^{1}H$) and 75 MHz ($^{13}C$); NEO600 Bruker spectrometer at 600 ($^{1}H$) and 243 ($^{31}P\{^{1}H\}$) MHz. Spectra are reported in δ (parts per million) relative to residual proteo-solvent signals for $^{1}$ H and $H_3PO_4$ (δ 0.00 ppm) for $^{31}P\{^{1}H\}$. The following abbreviations were used to explain multiplicities: s = singlet, d = doublet, t = triplet, q = quartet, m = multiplet. Deuterated solvents were purchased from Cambridge Isotope Laboratories and used as received for all NMR experiments.

Biotage column chromatography

Column chromatography was performed on a Biotage Isolera One 3.0 autocolumn instrument using KP-Sil high-performance columns repacked using Silicycle silica (P60, particle size 40–63 μm, column sizes described in experimental). TLC was performed using Millipore Sigma silica plates (60F-254) using short-wave UV light as visualizing agents. Electrospray ionization (ESI) mass spectra were obtained using an Agilent 6530 QTOF-ESI in tandem with a 1260 Infinity LC. DART mass spectra were obtained using a Thermo Exactive Plus Orbitrap with IonSense IDCUBE DART source.

High-resolution mass spectrometry (HRMS)

High-resolution mass spectrometry data was collected using either an Agilent 1260 Infinity 6530 Q-TOF ESI instrument (ESI/QTOF), a Waters LCT premier mass spectrometer with a direct inject port (ESI), or a Thermo Exactive Plus Orbitrap instrument with IonSense ID-CUBE DART source (DART).

Infrared Spectroscopy (IR)

IR spectra were obtained using PerkinElmer Spectrum ONE FT-IR Spectrometer.

Analytical reverse-phase high performance liquid chromatography (HPLC)

HPLC was carried out on a Agilent 1260 Infinity II HPLC system equipped with an autosampler and a UV detector using a Poroshell 120 2.7 μm C18 120 Å column (Analytical: 2.7 μm, 4.6 × 100 mm) with monitoring at λ = 220 and 280 nm and with a flow rate of 0.8 mL/min. Preparative HPLC purification was carried out on an Agilent 1290 Infinity II liquid chromatography system equipped with a UV detector using a Luna 5 μm C18 100 Å column (Preparatory: 5 μm, 250 × 21.2 mm) with monitoring at λ = 214 and 254 nm and with a flow rate of 20 mL/min. Individual gradients for each product purified via reverse phase HPLC are specified in their respective procedures.

Fast protein liquid chromatography (FPLC)

T4-Lysozyme (T4L) and T4-Lysozyme-Br (T4L-Br) were purified by FPLC on a Bio-Rad chromatography system. All purifications were carried out at 4 °C. All buffers were freshly prepared and filtered over a 0.2 μm PTFE filter prior to use.

Analytical FPLC analysis was conducted on a Bio-Rad Enrich SEC 650 10 × 300 Column. PBS buffer (20 mM phosphate, pH 6.5 with 150 mM NaCl) was freshly prepared and filtered over a 0.2 µm PTFE filter prior to use.

Liquid chromatography-mass spectrometry (LC-MS)

LC-MS analysis was carried out using an Agilent 6530 ESI-Q-TOF. DARPin analyses were carried out using an Agilent ZORBAX 300SB C3 column (3.5 µm, 3.0 × 150 mm). Liquid chromatography method used for proteins: Column temperature: 40 °C. Flow rate: 0.5 mL/min. Gradient: 90% water (0.1% formic acid (FA)) for 2 min; 90%-9% water (0.1% FA) 2-11 min; 5% water (0.1% FA) from 11-12 min. Sample preparation: For 70 µM reactions, 10 µL was added to 90 µL of 50:50 MeCN:$H_2O$ with 0.1% formic acid. For 7 µM reactions, 50 µL was added to 50 µL of 50:50 MeCN:$H_2O$ with 0.1% formic acid.

Size-exclusion Chromatography (SEC)

DMF SEC was conducted on an Agilent 1260 Infinity II high performance liquid chromatography (HPLC) system with a Wyatt Optilab (RI and MALS detection), one Polymer Laboratories PLgel guard column, and two Polymer Laboratories PLgel 5 µm mixed D columns. The eluent was DMF (HPLC Grade, 99.7+%, Thermo Scientific Chemicals) containing LiBr (0.1 M) at 40 °C (Flow rate: 0.6 mL/min). Molecular weight information was determined for data collected using a PMMA (Agilent Technologies, EasiVial PMMA, preweighed calibration kit) conventional calibration analysis.

Aqueous SEC was conducted on a Shimadzu Nexera 40 Series high performance liquid chromatography (HPLC) system equipped with a Shimadzu refractive index detector (RID-20A) and one shodex OHpak LB-806M column operating at 25 °C. Analyses were conducted in PBS (20 mM phosphate, pH 5.0 with 500 mM NaCl) at a 1.0 mL/min flow rate. Columns were calibrated with narrow molecular weight distribution Shodex P-82 standards with a range of 5000 – $10^6$ g $mol^{-1}$.

Sodium dodecyl sulfate polyacrylamide gel electrophoresis (SDS-PAGE)

Samples were dissolved in SDS-PAGE loading buffer containing 5% (v/v) 2-mercaptoethanol and 0.5% (m/v) bromophenol blue. After heating at 95 °C for 5 min, the samples were loaded onto a precast polyacrylamide gel. The gel was run on a Mini-Protein gel system (Bio-Rad) at 195 V for 90 min in running buffer (25 mM Tris, 250 mM glycine, and 0.1% SDS), followed by Coomassie Blue staining.

**Synthesis of an Aryl Iodide ATRP Initiator Precursor (1)**

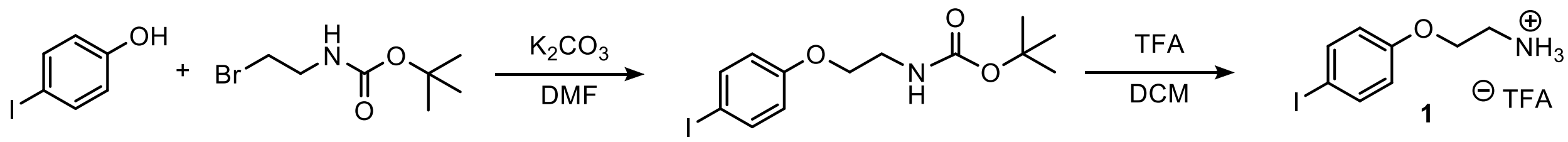

To a 100 ml round bottom flask charged with a stir bar was added 4-iodophenol (2.00 g, 9.09 mmol, 1 eq) and DMF (30 ml). To this solution, potassium carbonate (7.54 g, 54.5 mmol, 6 eq) was added under stirring and the solution was allowed to stir for 30 min. Then, *tert*-butyl (2-bromoethyl)carbamate (2.44 g, 10.9 mmol, 1.2 eq) was added. The reaction was heated to 80 °C and stirred for 14 hours. The reaction was then allowed to cool to ambient temperature, diluted with water, and extracted with hexanes (3 x 100 ml). The combined organic layers were dried over anhydrous $Na_2SO_4$ and then concentrated under reduced pressure to afford a clear and colorless liquid. This liquid was then dissolved in DCM (8 ml) in a scintillation vial equipped with a stir bar. To this vial was slowly added trifluoroacetic acid (2 mL, 0.03 mol, 20 eq), resulting in the evolution of gas. The reaction was then stirred for 3 hours. Volatiles were removed under reduced pressure and the product was triturated in diethyl ether (3 ml) thrice resulting in a crystalline white solid (1.52 g, 76% yield over two steps).

**$^1$H NMR (300 MHz, $CD_3CN$):** δ 7.65 – 7.59 (m, 2H), 6.81 – 6.76 (m, 2H), 4.19 – 4.16 (m, 2H), 3.34 – 3.31 (m, 2H).

**$^{13}$C NMR (101 MHz, $CD_3CN$):** δ 161.75, 159.06, 139.35, 83.98, 64.99, 40.06.

**HRMS (ESI):** $[M\text{-}TFA^-]^+$ calculated $C_8H_{11}INO^+$ 263.9885, observed 263.9824.

**IR (Film)**: 3427, 3082, 2891, 1584, 1485, 1466, 1274, 1239, 1182, 1089, 1043 $cm^{-1}$.

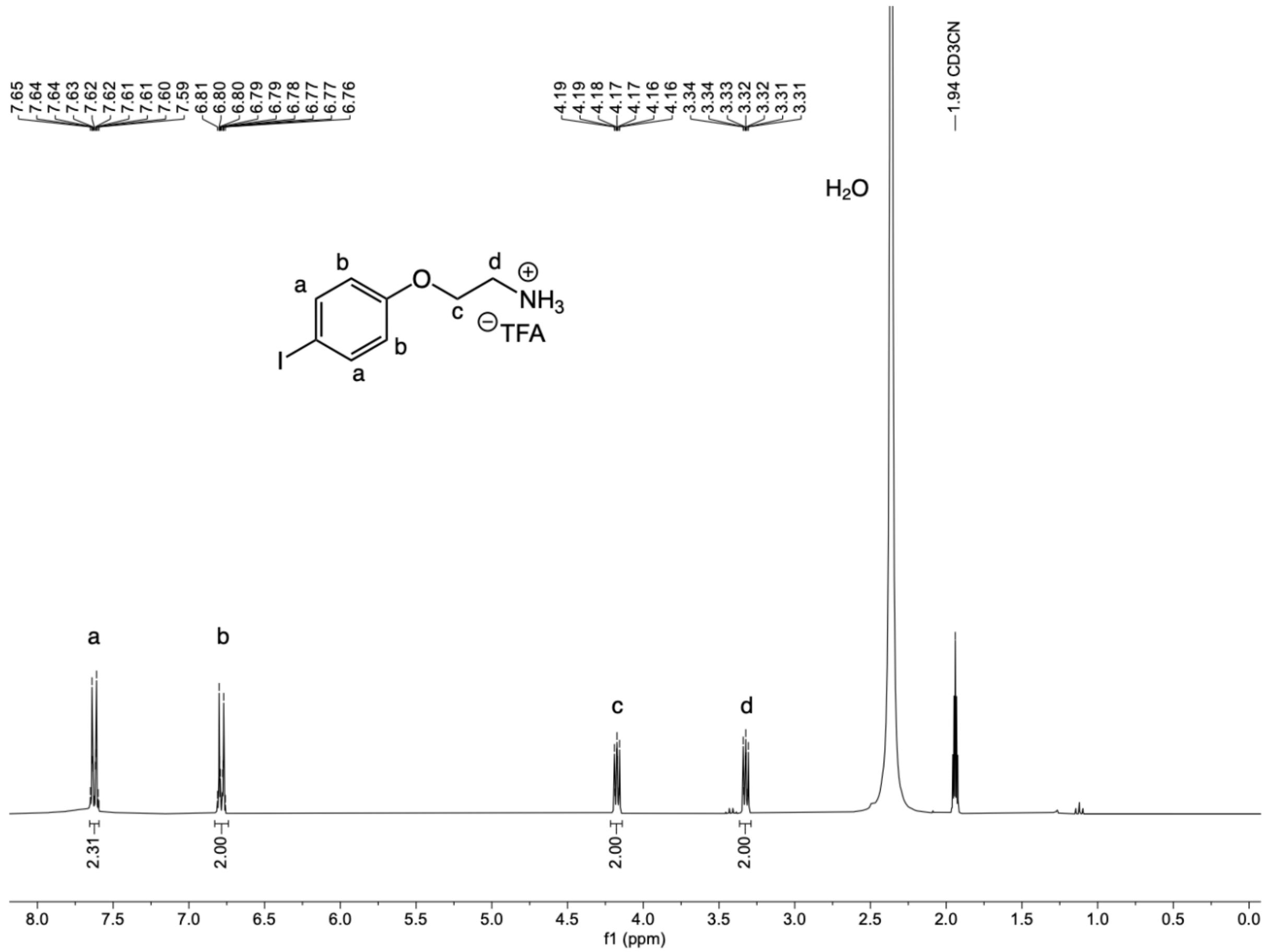


**Figure S1.** $^1$H NMR spectrum of 2-(4-iodophenoxy)ethanaminium TFA in $CD_3CN$ at 25 °C.

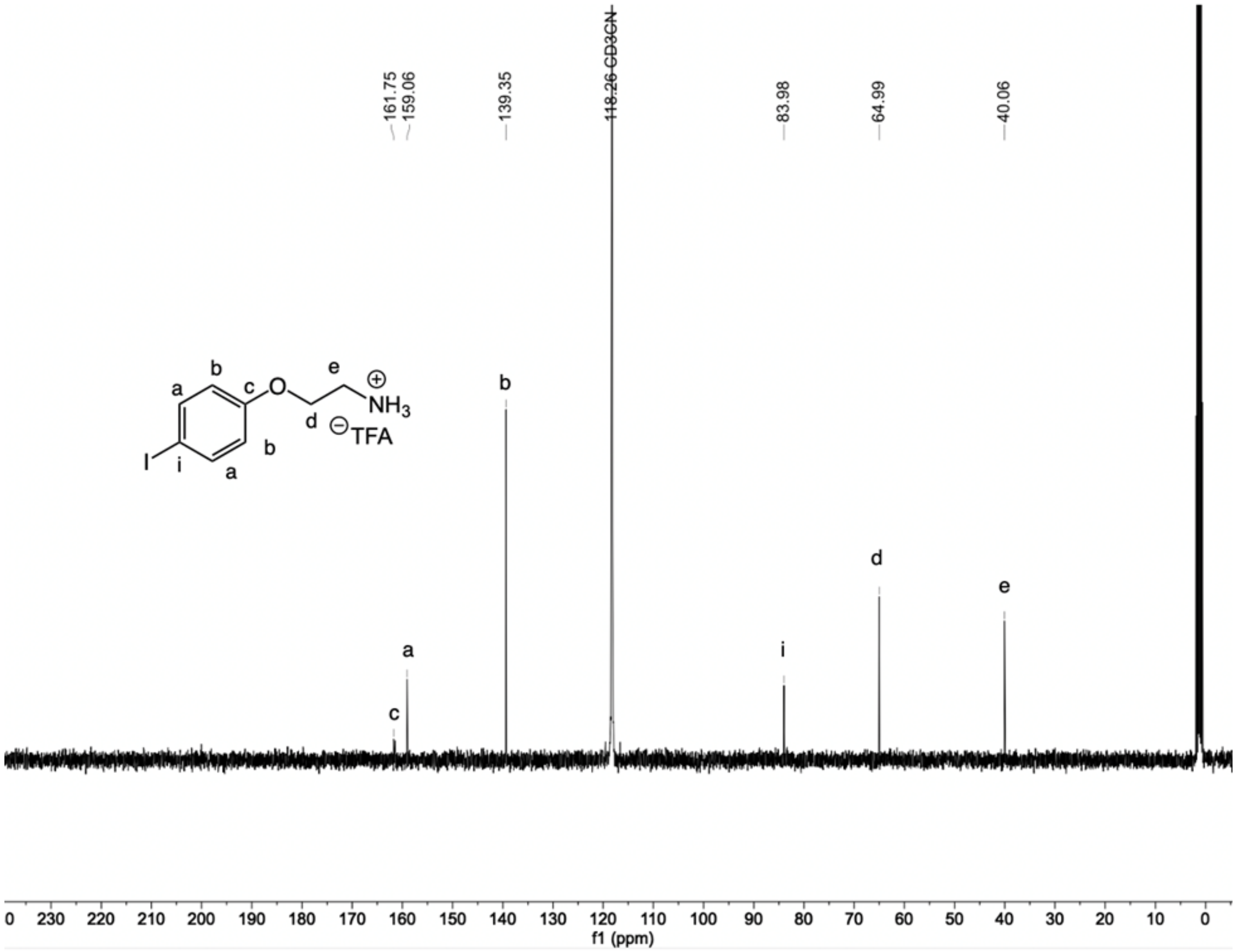


**Figure S2.** $^{13}$C NMR spectrum of 2-(4-iodophenoxy)ethanaminium TFA in $CD_3CN$ at 25 °C.

**Synthesis of an Aryl Iodide ATRP Initiator (2)**

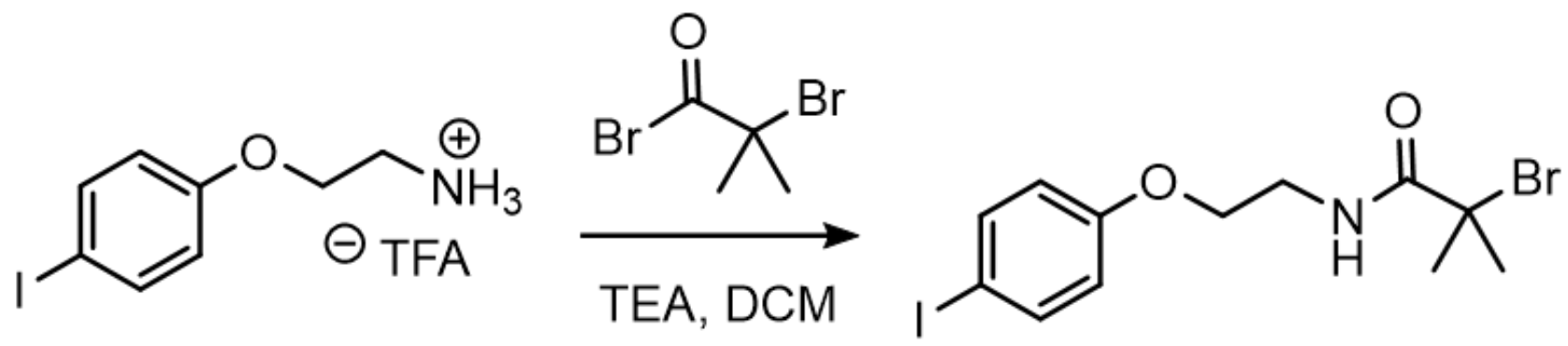


**2**

To a round bottom flask charged with a stir bar was added 2-(4-iodophenoxy)ethan-1-amine 2,2,2-trifluoroacetate (400 mg, 1.06 mmol, 1 eq) and DCM (8 mL) resulting in a white suspension. TEA (537 mg, 739 µL, 5.30 mmol, 5 eq) was added, and all solids were completely dissolved. This solution was allowed to stir for five min after which 2-bromo-2-methylpropanoyl bromide (732 mg, 423 µL, 3.18 mmol, 3 eq) was added dropwise resulting in the evolution of gas and a slight exotherm. This reaction was then allowed to stir at room temperature for two hours. After two hours, the reaction mixture was diluted in DCM and rinsed with water once, then brine. The organic layer was dried over magnesium sulfate and volatiles were removed under reduced pressure. The resulting oil was subjected to silica column chromatography in 0-100% EtOAc. The product eluted first, providing a white solid (389 mg, 89% yield). The product can be further purified by recrystallization from hexanes if small impurities remain.

**$^{1}$H NMR (300 MHz, $CDCl_3$):** δ 7.59 – 7.54 (m, 2H), 7.12 (s, 1H), 6.72 – 6.67 (m, 2H), 4.06 – 4.02 (m, 2H), 3.67 (td, *J* = 5.7, 4.8 Hz, 2H), 1.95 (s, 6H).

**$^{13}$C NMR (101 MHz, $CDCl_3$):** δ 172.47, 158.53, 138.51, 117.15, 83.57, 66.70, 62.84, 39.92, 32.67.

**HRMS (ESI):** $[M+H]^+$ calculated $C_{12}H_{15}BrINO_2^+$ 411.9401, observed 411.9409.

**IR (Film)**: 3012, 2982, 1683, 1588, 1485, 1479, 1388, 1370, 1269, 1136, 1106, 1084 $cm^{-1}$.

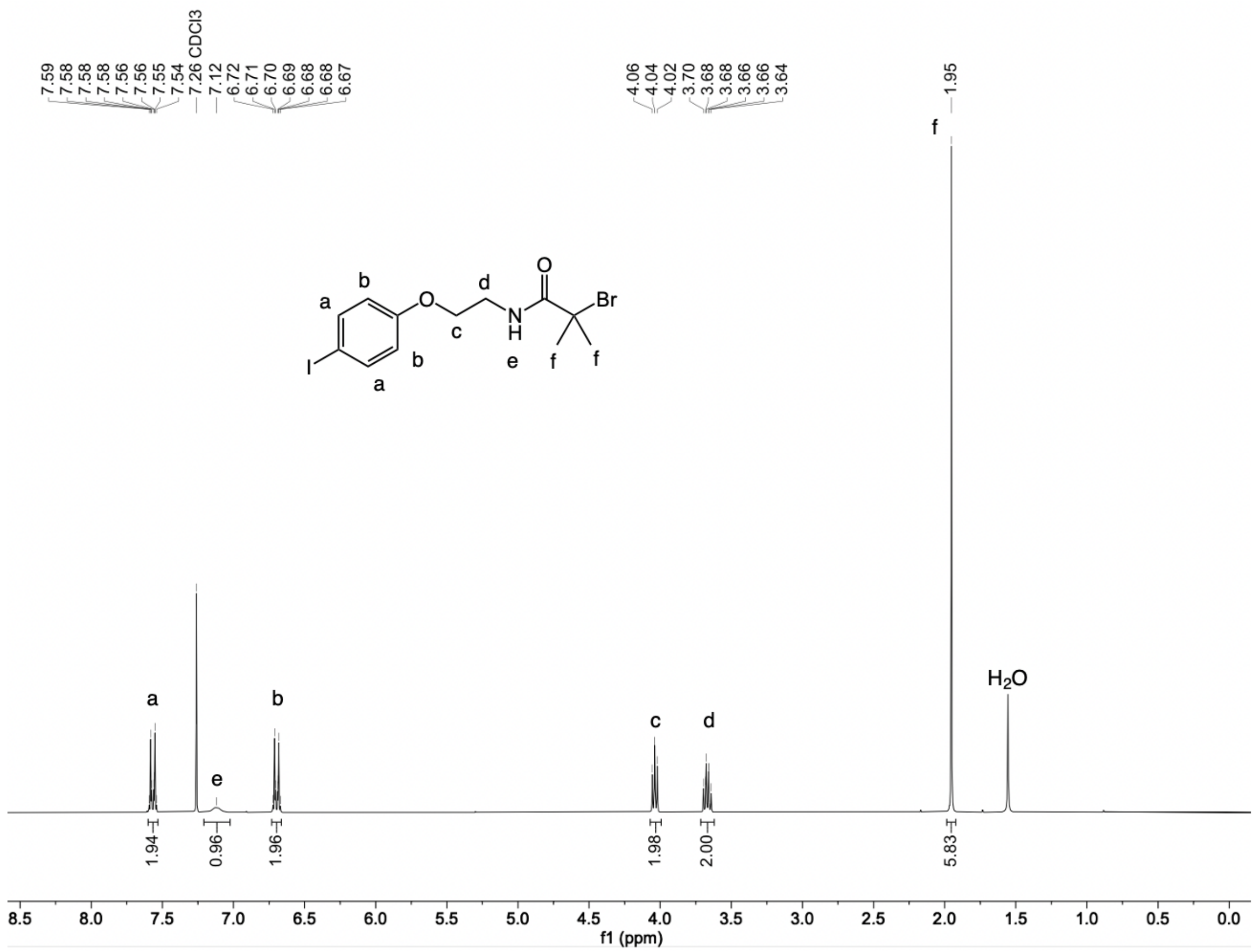


**Figure S3.** $^1$H NMR spectrum of ArI-ATRP initiator **(2)** in $CDCl_3$ at 25 °C.

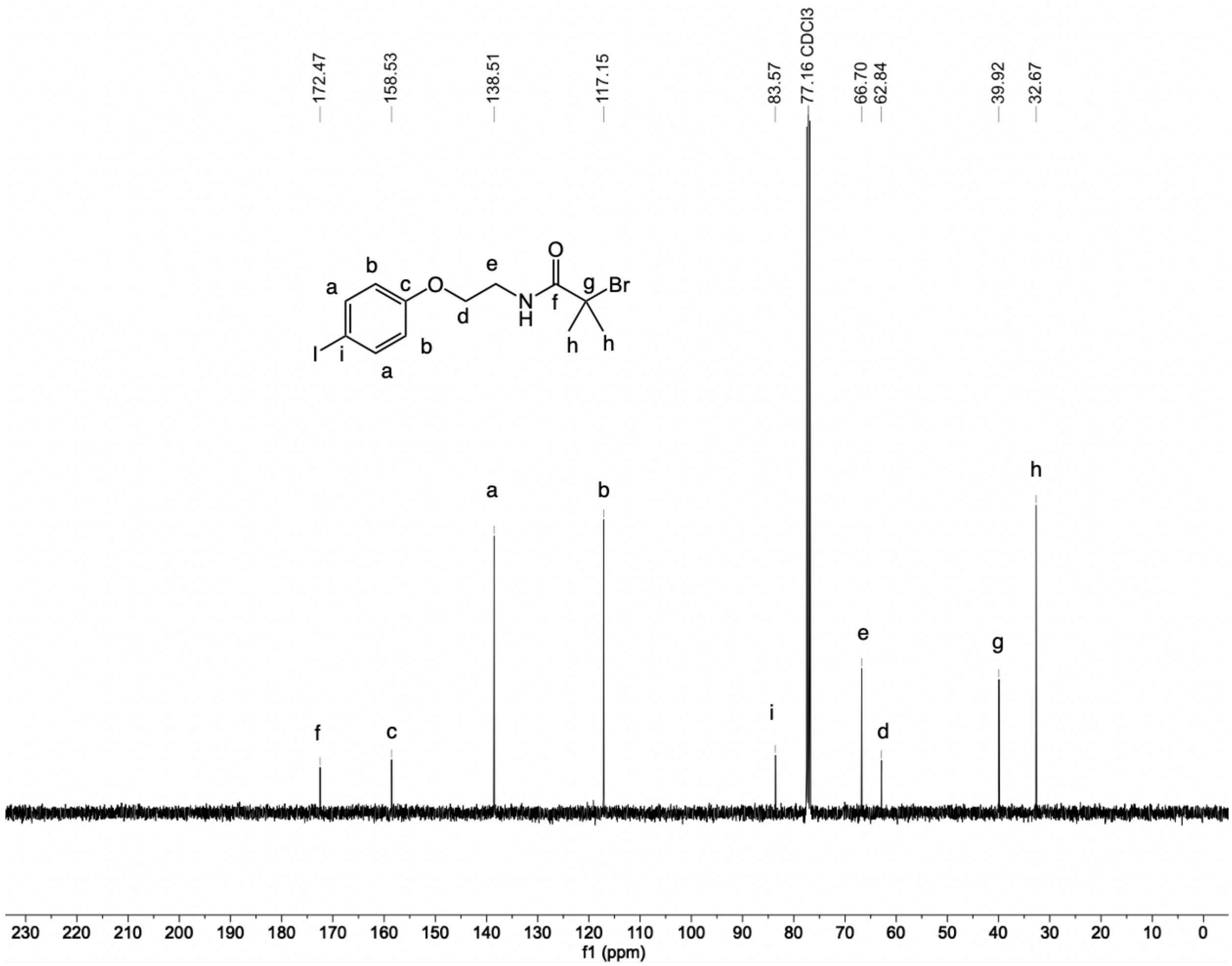


**Figure S4.** $^{13}C$ NMR spectrum of ArI-ATRP initiator **(2)** in $CDCl_3$ at 25 °C.

**Synthesis of an Aryl Iodide ATRP Initiator (2')**

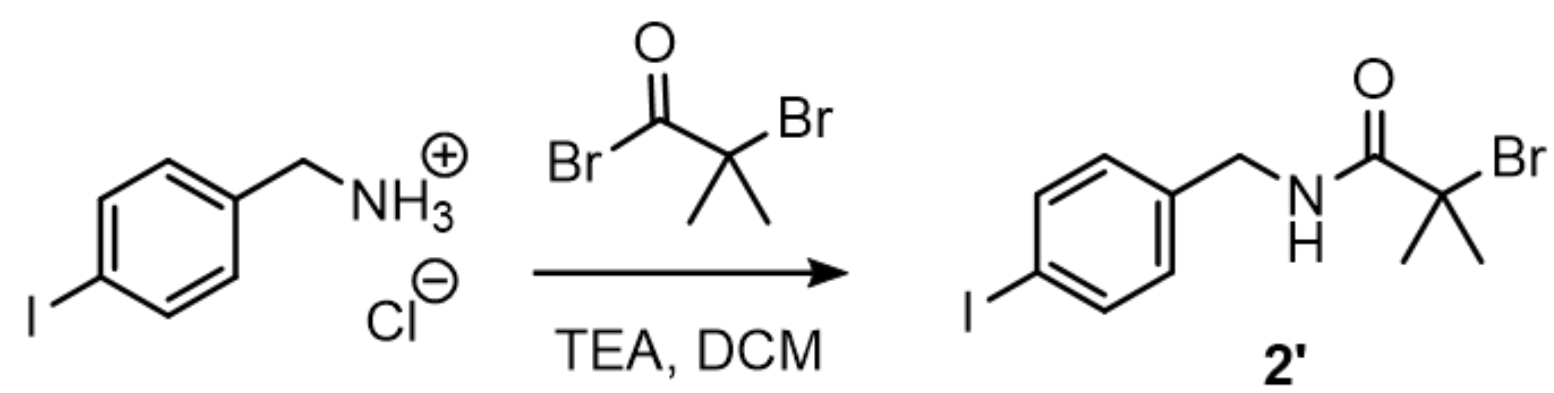


2'

To a 250 mL round-bottom flask charged with a magnetic stir bar, 4-iodobenzylamine hydrochloride (2.00 g, 7.42 mmol, 1.0 eq.) and anhydrous DCM (150 mL) were added. To this solution, TEA (2.63 g, 25.97 mmol, 3.5 eq.) was added under stirring, and the mixture was cooled in an ice bath. 2-Bromo-2-methylpropanoyl bromide (2.05 g, 8.91 mmol, 1.2 eq.) was then added dropwise. The formation of white salts was observed during the addition, and the reaction mixture was vigorously stirred for 18 h at room temperature. After completion, the white precipitate was removed by filtration. The filtrate was diluted with DCM and washed sequentially with 1 M HCl solution (3 x 250 mL), saturated aqueous $NaHCO_3$ (3 x 250 mL), and brine (3 x 250 mL). The organic layer was dried over anhydrous $MgSO_4$, filtered, and concentrated under reduced pressure. The crude product was purified by flash column chromatography (Biotage) using an isocratic eluent of EA:Hexane (1:4, v/v). The target compound **2'** was obtained as a white solid after drying in a vacuum oven overnight (Yield: 87%).

**$^1$H NMR (400 MHz, DMSO):** δ 8.75 (t, 1H), 7.81 (d, 2H), 7.16 (d, 2H), 4.52 (d, 2H), 1.94 (s, 6H).

**$^{13}$C NMR (101 MHz, $CDCl_3$):** δ 172.09, 137.87, 137.58, 129.49, 93.06, 62.72, 43.82, 32.59

**HRMS (ESI):** $[M+H]^+$ calculated $C_{11}H_{14}BrINO^+$ 383.9285, observed 383.9424.

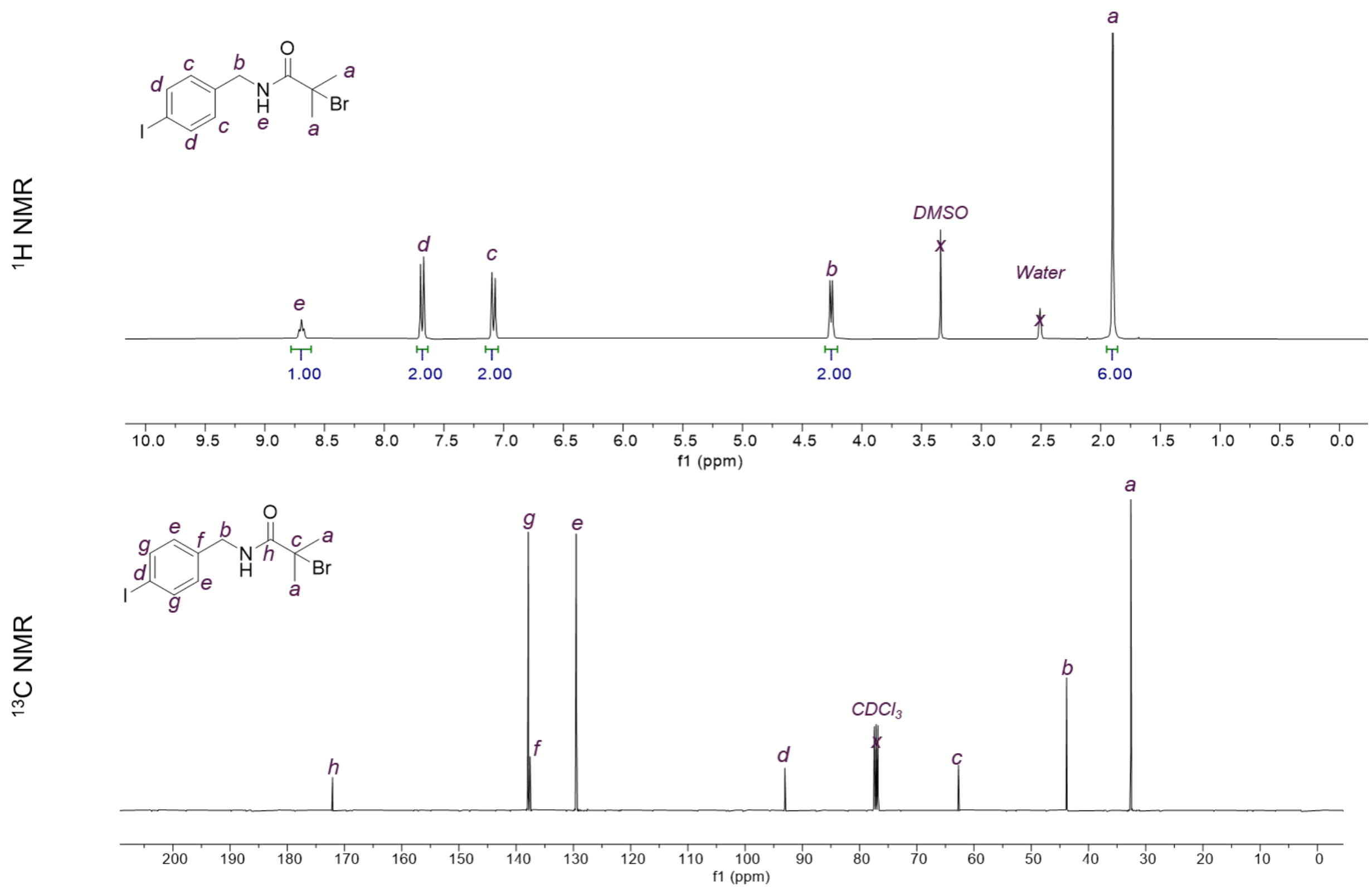


**Figure S5.** $^{1}H$ and $^{13}C$ NMR spectra of Aryl Iodide ATRP Initiator (**2’**) in DMSO-$d_6$ ($^{1}H$) and $CDCl_3$ ($^{13}C$) at 25°C.

**Synthesis of an Aryl Iodide ATRP Initiator (3)**

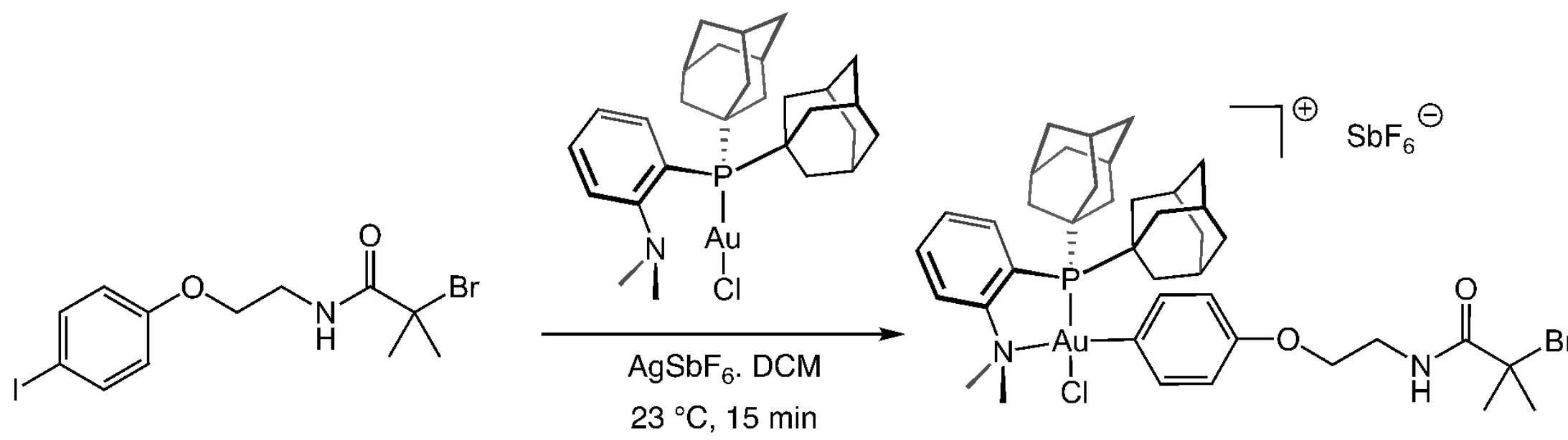


Upon removal from a nitrogen atmosphere glove box, $AgSbF_6$ (20 mg, 0.058 mmol, 1 eq) was dissolved in DCM (2 mL) under protection from light, and the solution was cooled to -20 °C. A DCM solution (2 mL) containing 2-bromo-N-(2-(4-iodophenoxy)ethyl)-2-methylpropanamide (36 mg, 0.087 mmol, 1.5 eq) and (MeDalPhos)AuCl (38 mg, 0.058 mmol, 1 eq) was prepared and also cooled to -20 °C. While both solutions were cold, the colorless aryl iodide and Au(I) solution was added in one portion to the solution of $AgSbF_6$, resulting in visible precipitation of solids and a color change from clear to bright yellow. The reaction was stirred for 15 min at 23 °C. Then, the reaction mixture was filtered through a pad of Celite to remove Ag salts. The solution was concentrated under reduced pressure and triturated in diethyl ether thrice to provide a bright yellow solid (67 mg, quantitative yield).

**$^1$H NMR (300 MHz, $CDCl_3$):** δ 8.02 – 7.90 (m, 3H), 7.83 – 7.73 (m, 1H), 7.33 (d, *J* = 8.9 Hz, 2H), 7.14 (s, 1H), 6.91 (d, *J* = 8.9 Hz, 2H), 4.10 (t, *J* = 5.2 Hz, 2H), 3.70 (q, *J* = 5.4 Hz, 2H), 3.51 (s, 6H), 2.26 (d, *J* = 9.4 Hz, 7H), 2.12 (s, 13H), 1.98 (s, 6H), 1.75 (s, 12H).

**$^{31}$P{$^1$H} NMR (121 MHz, $CDCl_3$):** δ 75.25 ppm.

**HRMS (ESI):** $[M]^+$ calculated $C_{40}H_{55}AuBrClN_2O_2P^+$ 937.2538, observed 937.2441.

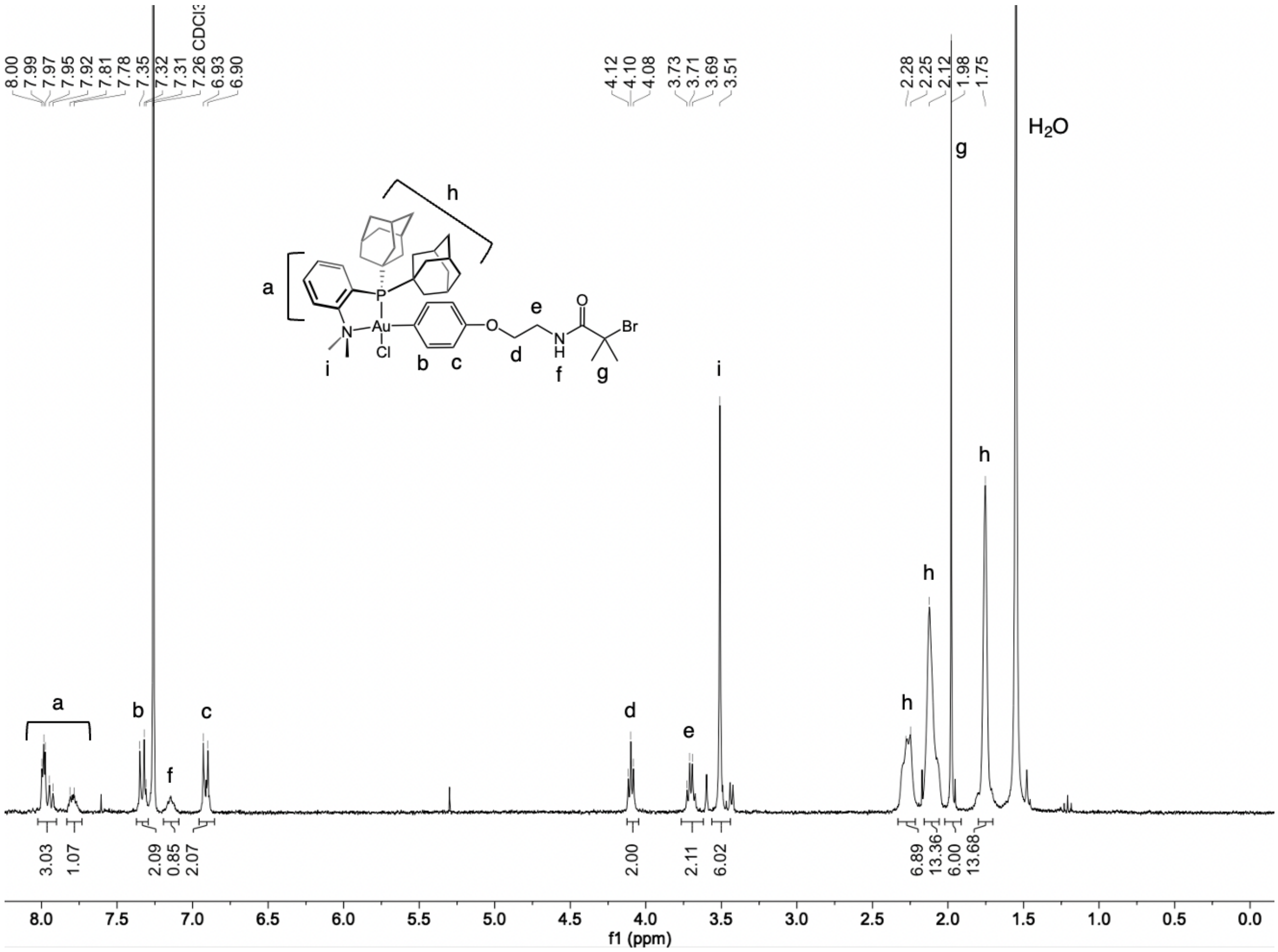


**Figure S6.** $^1$H NMR spectrum of Au(III)-ATRP initiator in $CDCl_3$ at 25 °C.

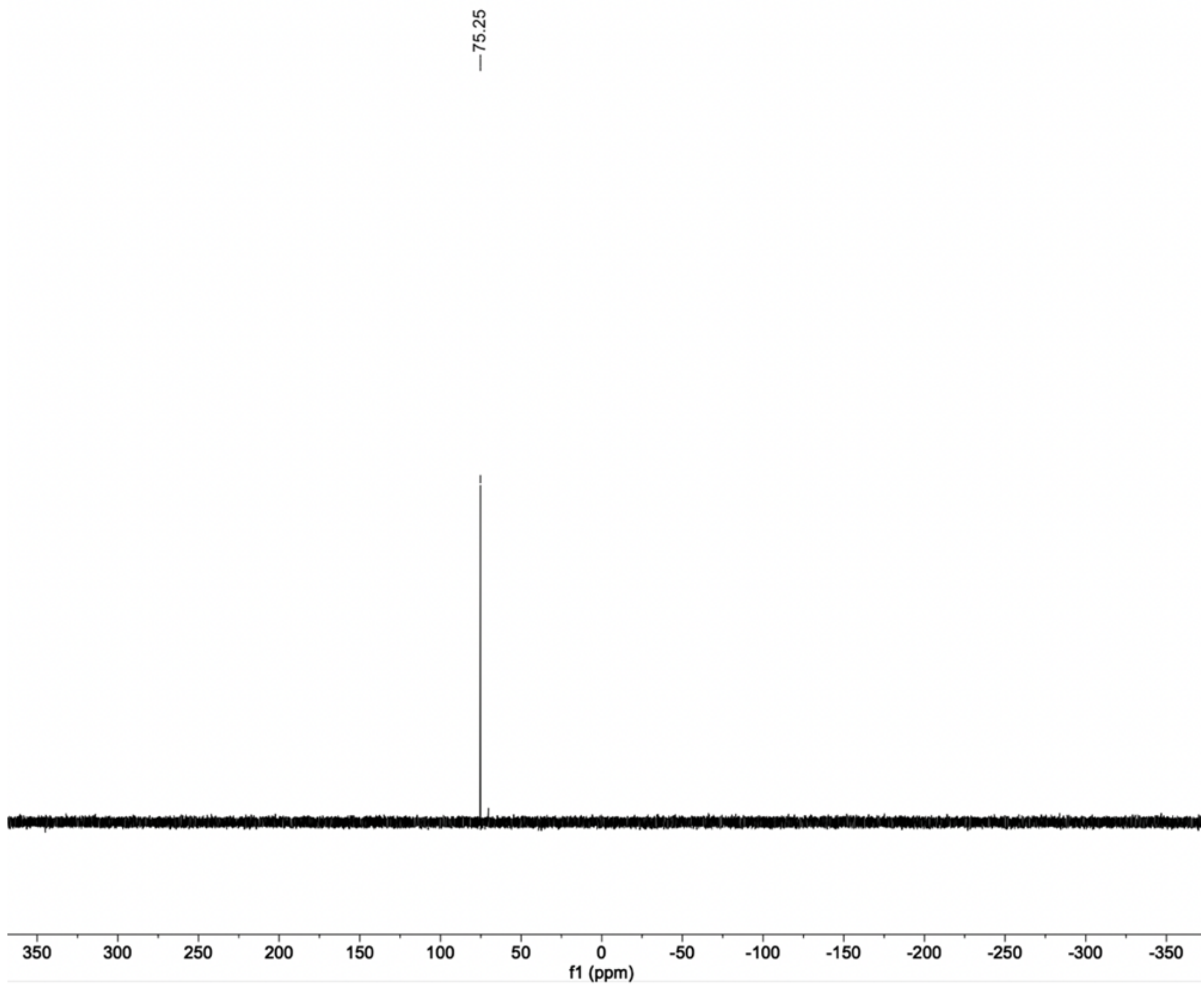


**Figure S7.** $^{31}P\{^1H\}$ NMR spectrum of Au(III)-ATRP initiator in $CDCl_3$ at 25 °C.

**General ATRP procedure** [1]

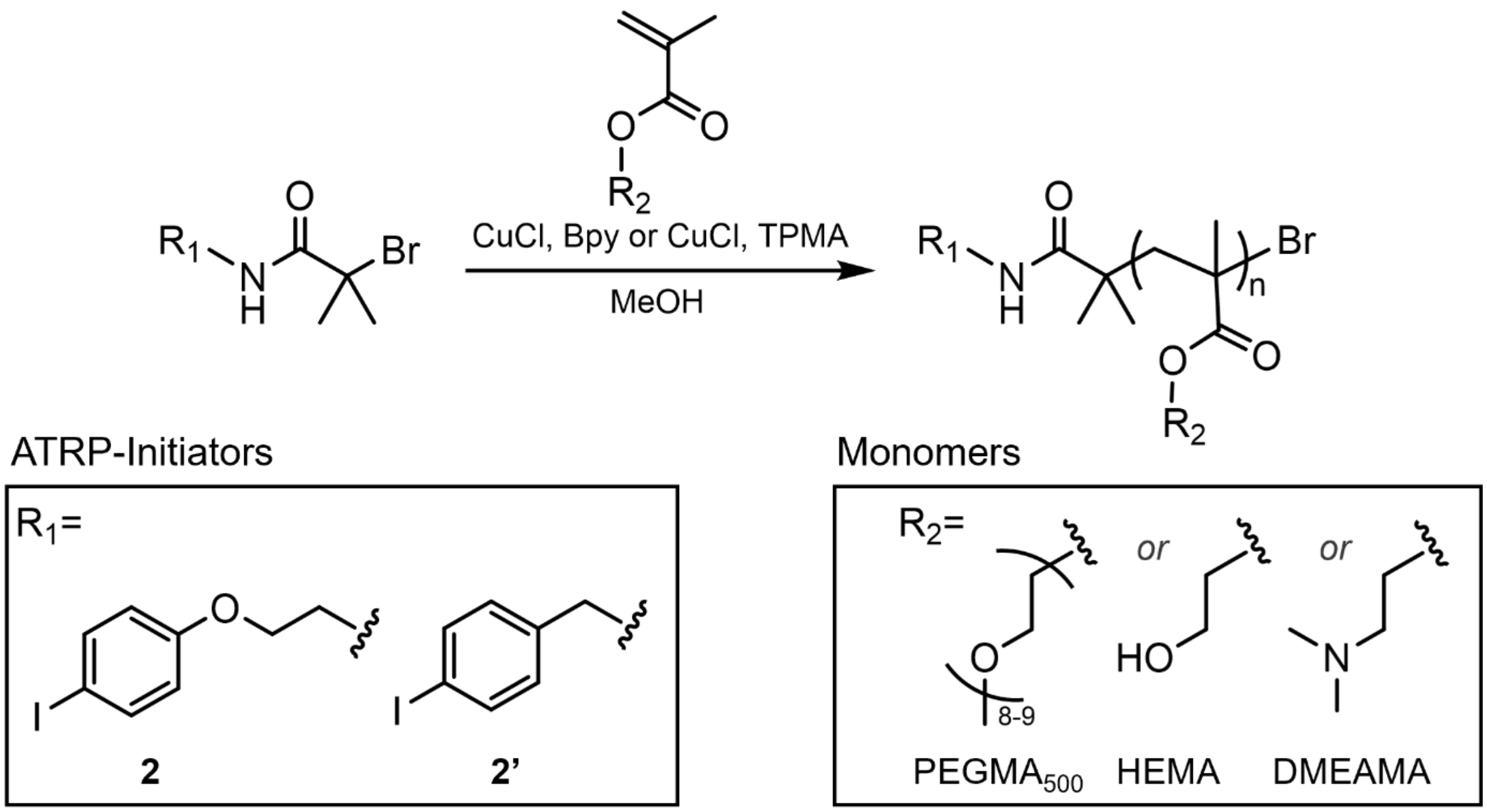

**Synthesis of pPEGMA-ArI**

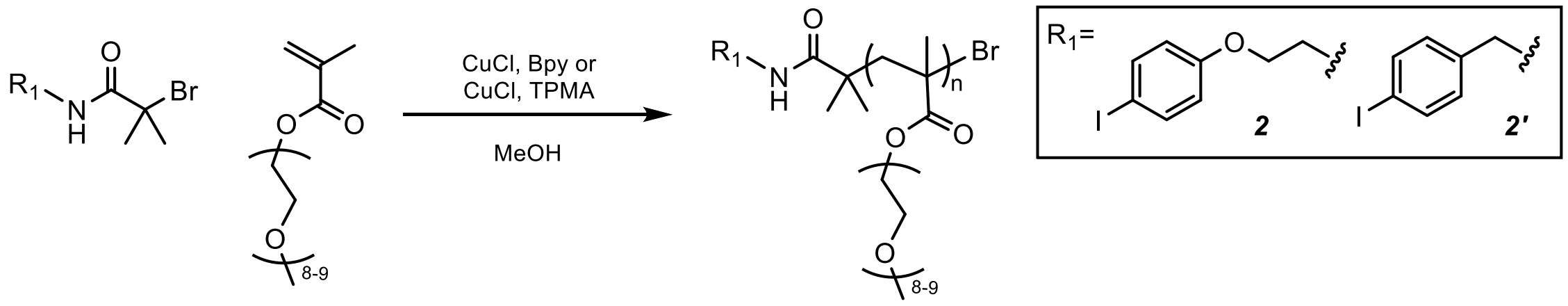


Initiator **2** (16 mg, 38.8 µmol, 1 equiv.) was dissolved in 500 µl of degassed methanol. Then, a catalyst solution was created by 2,2'-bipyridine (bpy) (12.1 mg, 77.7 µmol, 2 equiv.) in 1 ml of degassed MeOH (sparged with argon for 15 min). This solution was added to a vial containing Cu(I)Cl (3.8 mg, 38.8 µmol, 1 equiv.). To this solution was added degassed $PEGMA_{500}$ monomer (604 mg, 1.16 mmol, 30 equiv.) using a syringe and the solution was further degassed by sparging with argon 5 min, followed by the addition of the initiator solution to begin polymerization. The reaction solution became dark brown and after 30 min, THF (50 ml) was added to the reaction solution. After filtration, the THF solution of polymer was passed through an alumina column to remove residual catalyst. After THF removal under vacuum, the residue was precipitated into cold diethyl ether three times and dried under reduced pressure, affording the product as a clear oil (72% yield, containing trace residual methanol).

**$^1$H NMR (400 MHz, $CD_3CN$):** δ 7.58 (d, J = 8.2 Hz, 2H), 6.75 (d, J = 8.1 Hz, 2H), 6.57 (s,br 1H), 4.05 (s, 118H), 3.67 – 3.59 (m, br, 123H), 3.54 (s, br, 1653H), 3.48 – 3.43 (m, br, 124H), 3.28 (s, br, 169H), 1.92 – 1.66 (m, br, 100H), 0.92 (d, br, 173H).
**SEC:** $M_w^{theor}$ = 15.5 kDa, $M_n^{NMR}$ = 31.0 kDa, $M_n^{SEC}$ = 29.5 kDa, $M_w^{SEC}$ = 39.3 kDa, $Đ$ = 1.33.

Initiator **2'** (20 mg, 54.2 µmol, 1.0 equiv.) was charged into a 10 mL Schlenk flask, followed by the addition of degassed $PEGMA_{500}$ monomer (785 mg, 1.57 mmol, 30 equiv.). The flask was further degassed by purging with argon gas, and then deoxygenated MEOH (1.5 mL) was injected into the flask. Separately, a stock solution of CuCl/tris(2-pyridylmethyl)amine (TPMA) was prepared in methanol with a CuCl concentration of 26 mg/mL (containing 2.0 equiv. of TPMA relative to CuCl). This stock solution was degassed by bubbling with argon for 15 min in an ice bath. Subsequently, a portion of the CuCl/TPMA stock solution (199.33 µL, containing 54.2 µmol of CuCl [1.0 equiv. relative to initiator] and 108.4 µmol of TPMA) was injected into the Schlenk flask to initiate the polymerization. The reaction was allowed to proceed at 35 °C for 30 min, during which the mixture turned dark brown. After 30 min, the reaction was quenched and diluted by adding THF (50 mL). The resulting polymer solution was filtered and passed through an alumina column to remove the residual copper catalyst. The solution was concentrated under vacuum and precipitated into cold diethyl ether three times. The residual solvent was then removed under reduced pressure to afford the product as a clear oil (yield: 67%).

**$^1$H NMR (400 MHz, $CDCl_3$):** δ 7.64 (d, J = 8.2 Hz, 2H), 7.12 (d, J = 8.1 Hz, 2H), 6.59 (s,br 1H), 5.42 (s, 2H), 4.05 (s, 104H), 3.67 – 3.59 (m, br, 1790H), 3.54 (s, br, 150H)
**SEC:** $M_w^{theor}$ = 15.5 kDa, $M_n^{NMR}$ = 25.3 kDa, $M_n^{SEC}$ = 27.8 kDa, $M_w^{SEC}$ = 38.5 kDa, $Đ$ = 1.37.

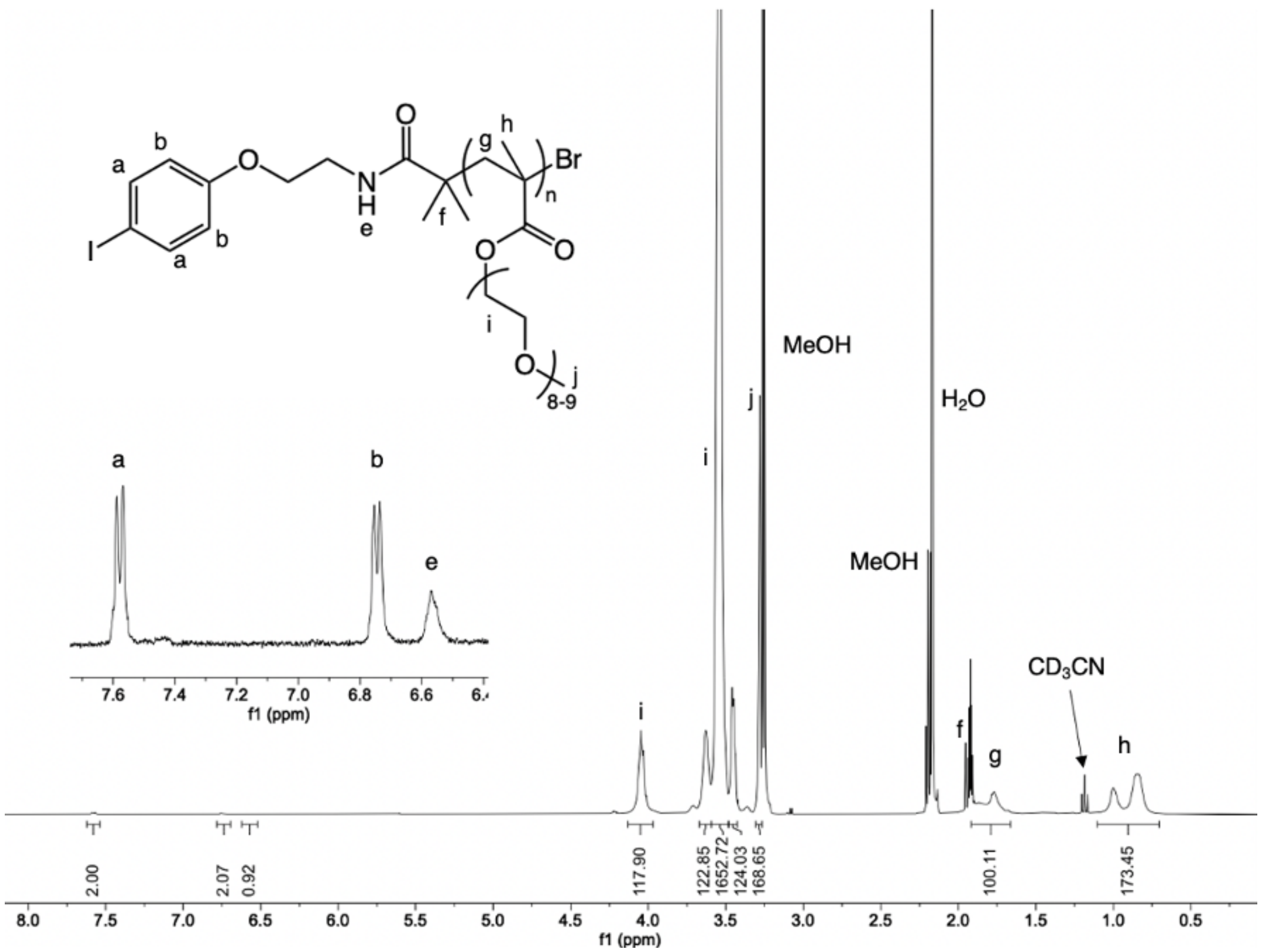


**Figure S8.** $^1$H NMR spectrum of pPEGMA-ArI in $CD_3CN$ at 25 °C.

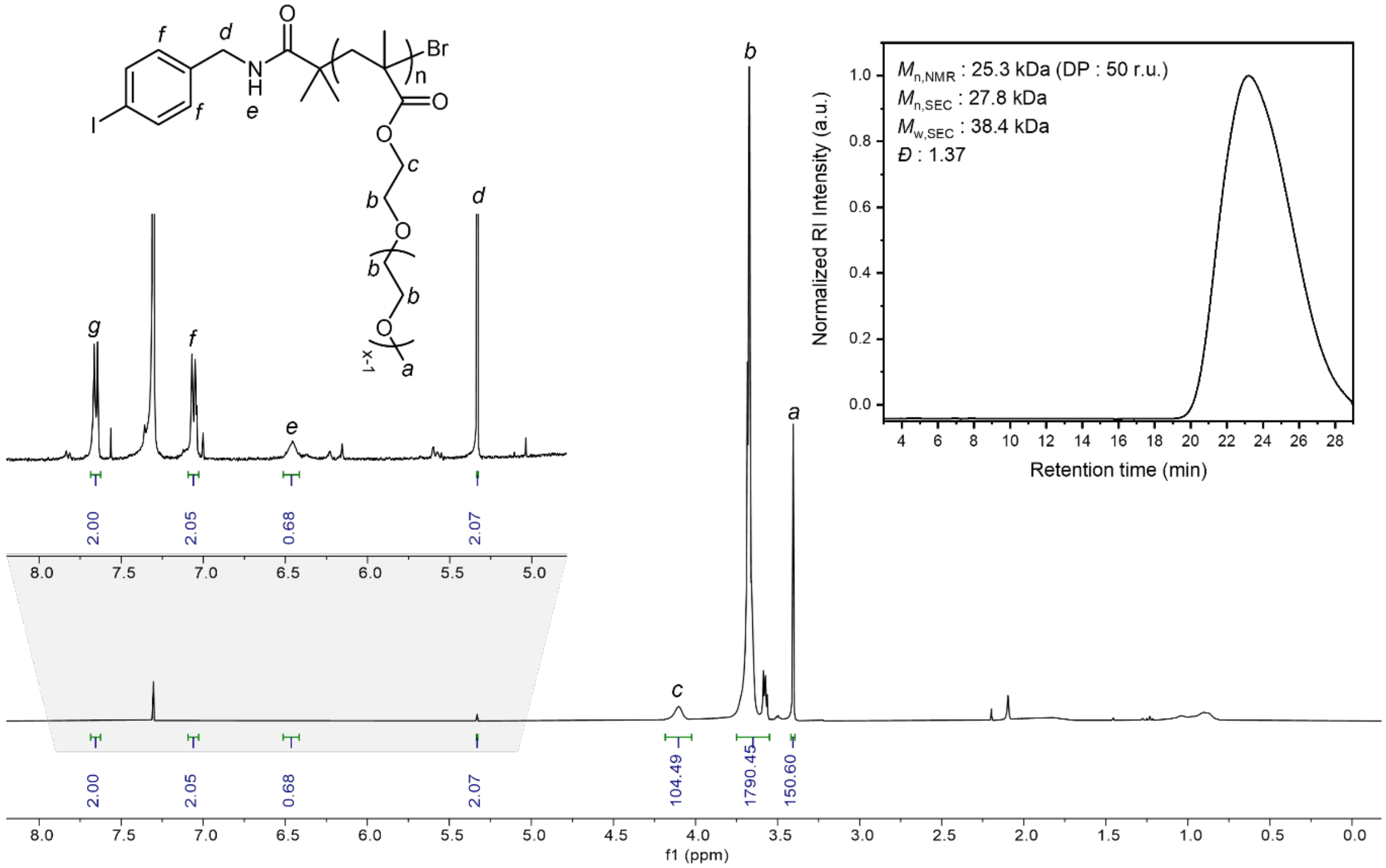


**Figure S9.** $^1$H NMR spectrum of pPEGMA-ArI synthesized using initiator **2'** (in $CDCl_3$ at 25 °C), with the DMF SEC trace shown as an inset.

**Synthesis of pHEMA-ArI**

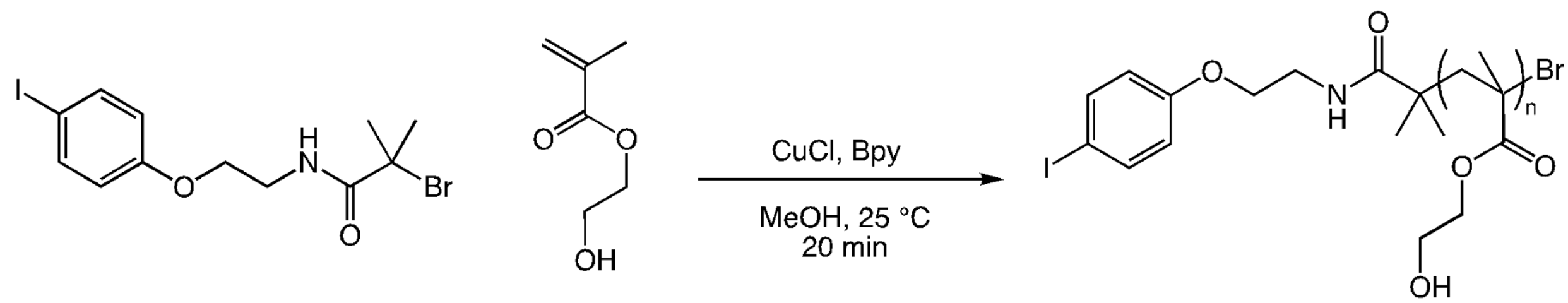


Initiator (12.6 mg, 30 μmol, 1 equiv.) was dissolved in 500 μl of degassed methanol. Then, a catalyst solution was created by dissolving 2,2'-bipyridine (bpy) (12.0 mg, 76.8 μmol, 2.5 equiv.) in 3 ml of degassed MeOH (sparged with argon for 15 min). This solution was added to a vial containing Cu(I)Cl (3.0 mg, 30.7 μmol, 1 equiv.). To this solution was added degassed HEMA monomer (400 mg, 3.07 mmol, 100 equiv.) using a syringe; the solution was further degassed by sparging with argon 5 min, followed by the addition of the initiator solution to begin polymerization. The reaction solution became dark brown and after 30 min, THF (50 ml) was added to the reaction solution, followed by excess $MgSO_4$ to absorb any water. After filtration, the THF solution of polymer was passed through an alumina column to remove residual catalyst. THF was then removed under vacuum, and the remaining solid was precipitated in diethyl ether three times, yielding product as white solid (yield 81%).

**$^1$H NMR (400 MHz, MeOD):** δ 7.15 (dd, J = 324.5, 8.3 Hz, 6H), 4.02 (s, 455H), 3.76 (d, J = 6.5 Hz, 477H), 1.96 (d, J = 26.4 Hz, 403H), 1.08 (s, 195H), 0.92 (s, 460H).

**SEC:** $M_n^{theor}$ = 13.0 kDa, $M_n^{NMR}$ = 29.2 kDa, $M_n^{SEC}$ = 48.4 kDa, $M_w^{SEC}$ = 78.8 kDa, $Đ$ = 1.63.

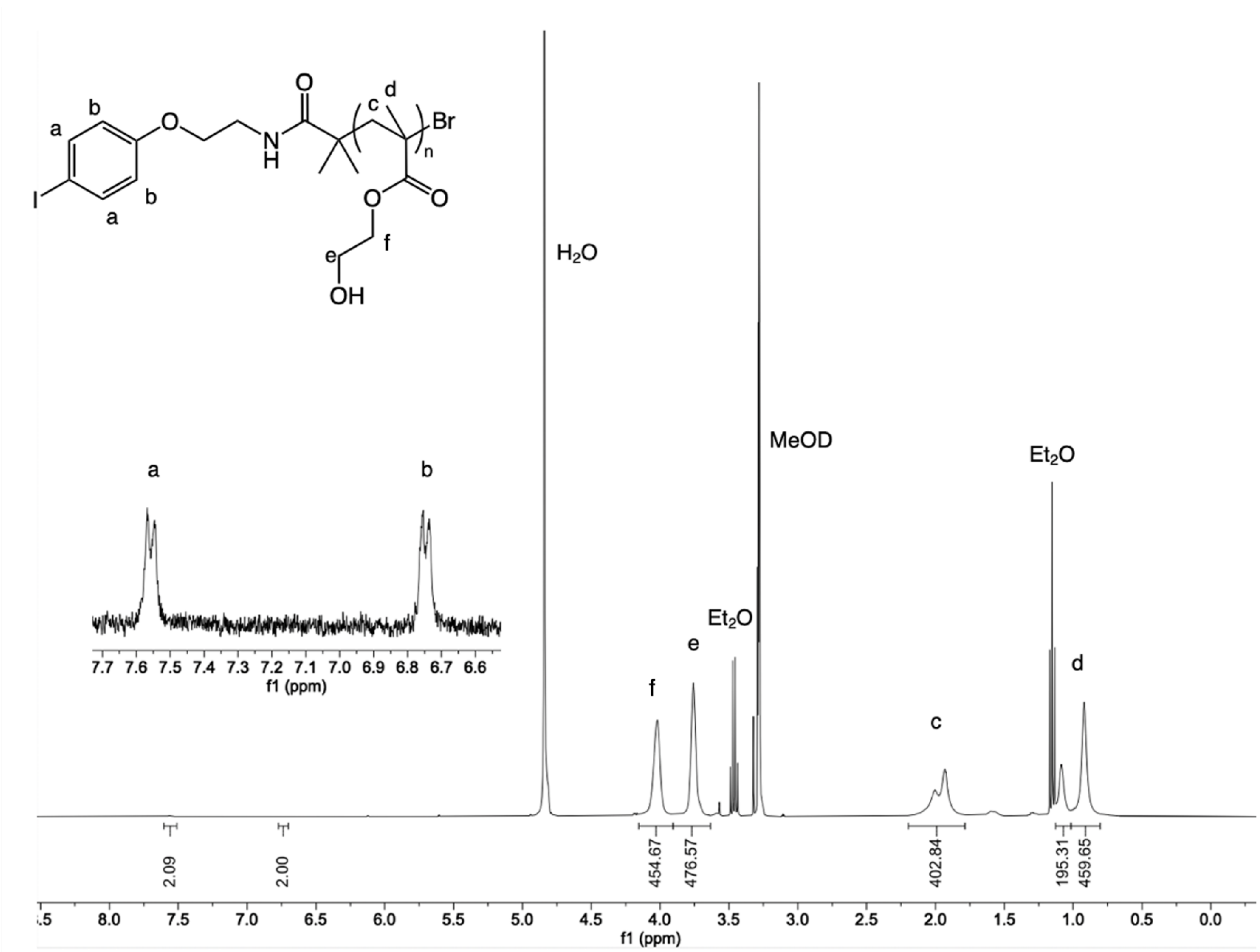


**Figure S10.** $^1$H NMR spectrum of pHEMA-ArI in MeOD at 25 °C

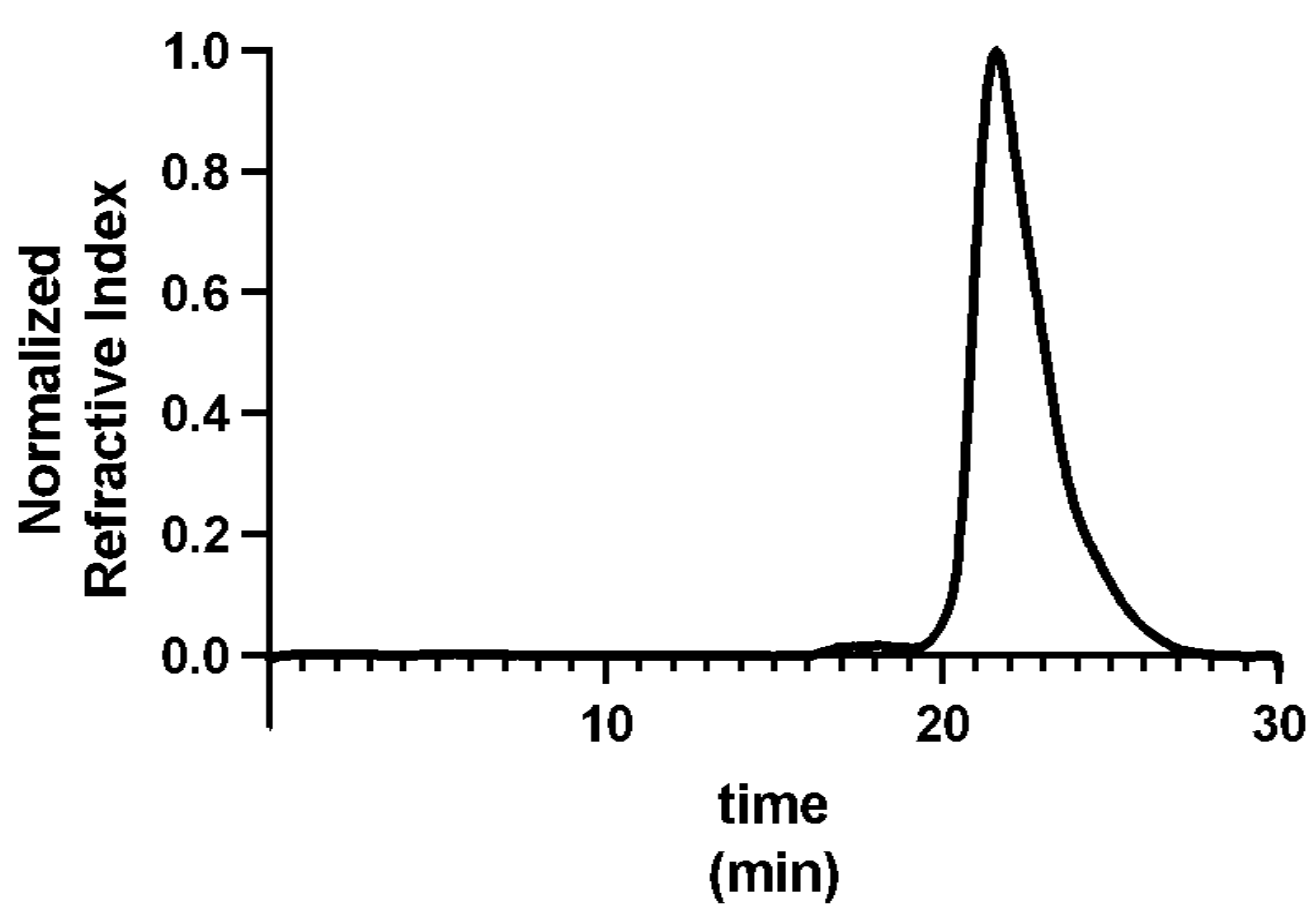


**Figure S11.** SEC data for polymer of pHEMA-ArI in DMF. (**Table 1, entry 2**)

**Synthesis of pDMAEMA-ArI**

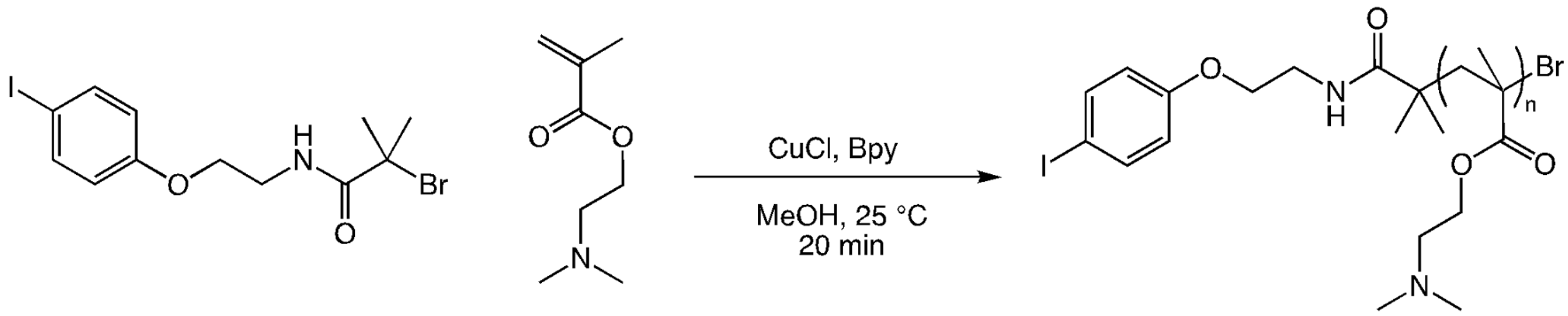


Initiator (10.5 mg, 25.4 μmol, 1 equiv.) was dissolved in 500 μl of degassed methanol. Then, a catalyst solution was created by dissolving 2,2'-bipyridine (bpy) (9.9 mg, 63.6 μmol, 2.5 equiv.) in 3 ml of degassed MeOH (sparged with argon for 15 min). This solution was added to a vial containing Cu(I)Cl (2.5 mg, 25.4 μmol, 1 equiv.). To this solution was added degassed DMAEMA monomer (400 mg, 2.54 mmol, 100 equiv.) using a syringe and the solution was further degassed by sparging with argon 5 min, followed by the addition of the initiator solution to begin polymerization. The reaction solution became dark brown and after 30 min, THF (50 ml) was added to the reaction solution, followed by excess $MgSO_4$ to absorb the water. After filtration, the THF solution of polymer was passed through an alumina column to remove residual catalyst. THF was then removed under vacuum, and the remaining solid was precipitated in diethyl ether three times, yielding product as white solid (yield 63%).

**$^1$H NMR (400 MHz, $CD_3CN$):** δ 7.75 (m, 2H), 6.8 (m, 2H), 4.02 (s, 148H), 2.63 (s, br, 149H), 2.24 (s, br, 544H), 1.81 (s, br, 77H), 0.8-1.01 (m, 222H).

**SEC:** $M_n^{theor}$ = 4.7 kDa, $M_n^{NMR}$ = 10.9 kDa, $M_n^{SEC}$ = 7.5 kDa, $M_w^{SEC}$ = 8.25 kDa, $Đ$ = 1.10.

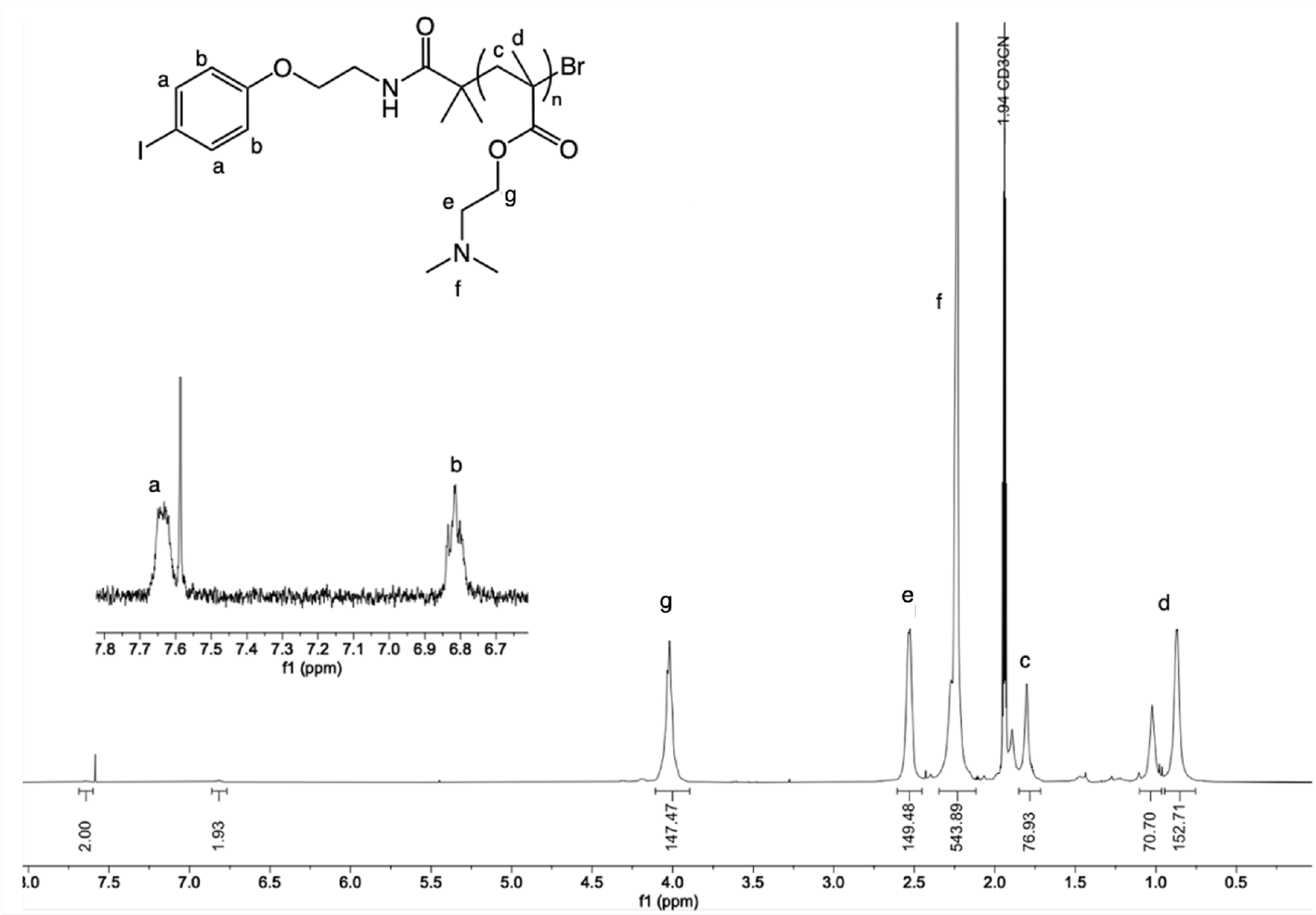


**Figure S12**: $^1$H NMR spectrum of pDMAEMA-ArI in $CD_3CN$ at 25 °C.

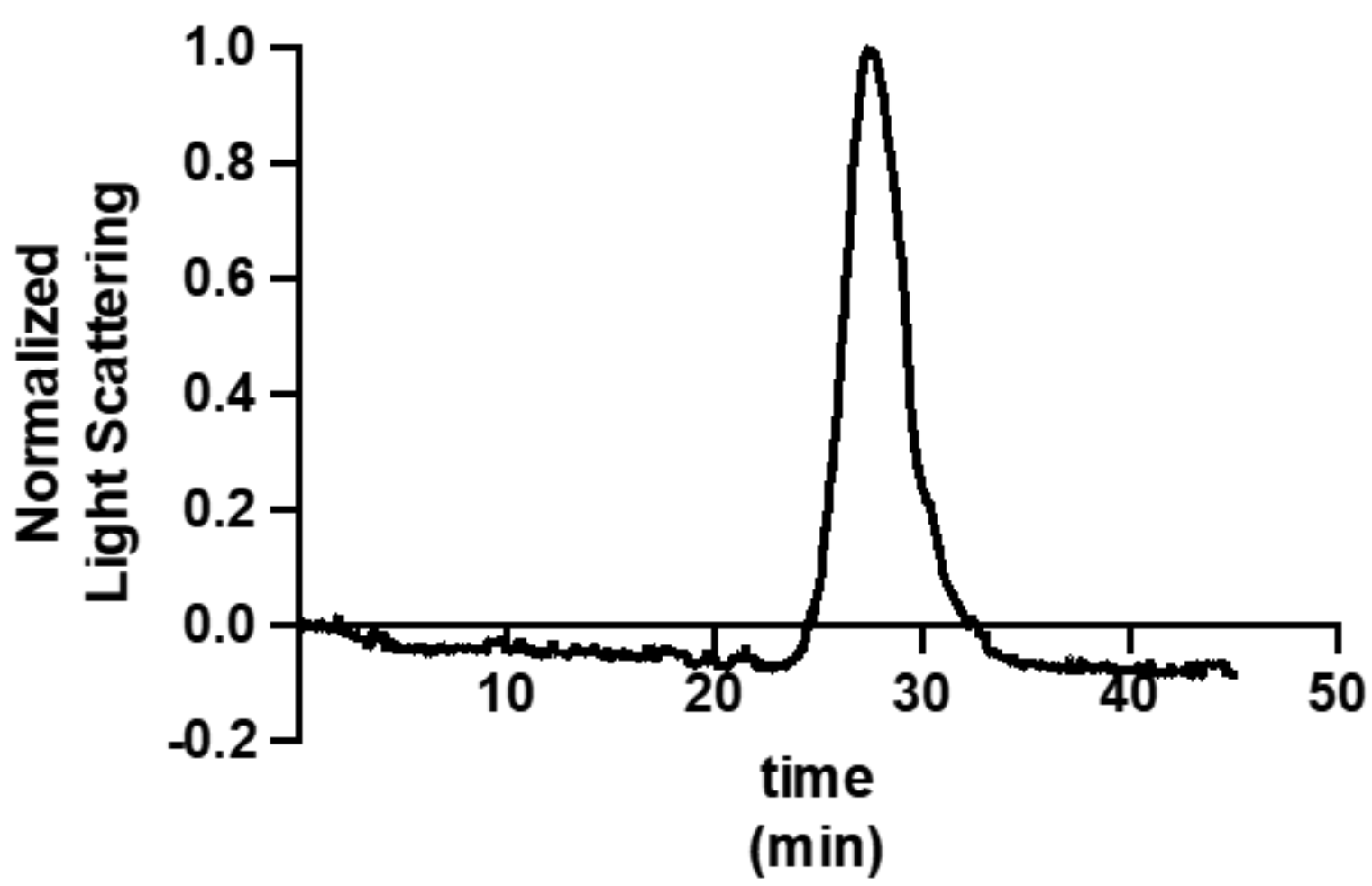


**Figure S13**: SEC data for polymer of pDMAEMA-ArI in DMF. (Table 1, entry 3)

**Synthesis of pPEGMA Au(III)-reagent**

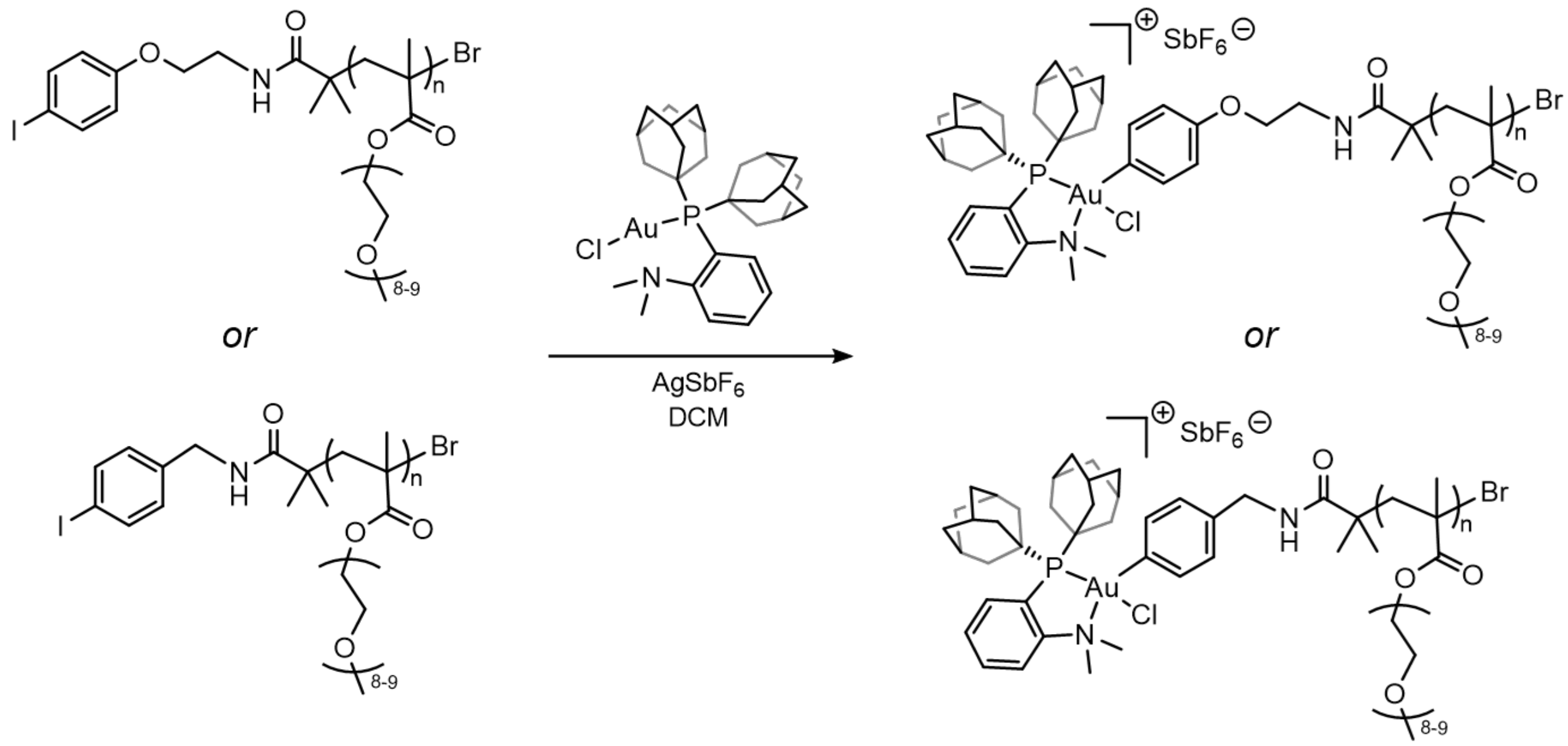

$AgSbF_6$ (13.3 mg, 3 equiv, 38.6 μmol) was dissolved in DCM (1 mL) under protection from light, and the solution was cooled to -20 °C. A DCM solution (1 mL) containing the pPEGMA-ArI (200 mg, 1 equiv, 12.9 μmol) and (Me-DalPhos)AuCl (12.6 mg, 1.5 equiv, 19.3 μmol) reagents was prepared and also cooled to -20 °C. While both solutions were cold, the colorless polymer and (Me-DalPhos)AuCl solution was added in one portion to the solution of $AgSbF_6$, resulting in visible precipitation of pale-yellow solids. The reaction was stirred for 14 hours at 25 °C. Then, the reaction mixture was filtered through a pad of Celite to remove Ag salts. The solution was concentrated under reduced pressure and used without further purification. The product was isolated as a pale yellow oil.

**$^{1}$H NMR (400 MHz, $CD_3CN$)** δ 7.91 (s, 1H), 7.72 (dd, *J* = 8.2, 6.9 Hz, 2H), 7.30 (d, *J* = 8.4, Hz, 2H), 6.81 (d, *J* = 8.5 Hz, 2H), 6.45 (s, 1H), 3.99 – 3.87 (s, br, 168H), 3.58 – 3.47 (m, 153H), 3.47 – 3.31 (m, 2290H), 1.50-2.00 (m, br, 30H), 0.89 (m, br, 139H). **(Figure 2c)**
**$^{31}$P{$^{1}$H} NMR (121 MHz, $CDCl_3$):** δ 77.13 ppm.

**Synthesis of Au(III) pPEGMA Reagent from Initiator 2':** The Au(III) pPEGMA reagent derived from initiator **2'** was synthesized following the exact same procedure described above. The final product was obtained as a pale-yellow oil. The material characterization data are presented in **Figure S14**.

**$^{1}$H NMR (400 MHz, $CDCl_3$)** δ 7.91 (t, 3H), 7.82 (t, 4H), 7.49 (d, 2H), 7.19 (d, 2H), 6.93 (s, 1H), 5.46 (s, 2H), 3.99 – 3.87 (s, br, 168H), 4.32 – 3.92 (m, 103H), 3.83 – 3.38 (m, 1549H), 1.14 – 0.79 (m, br, 144.2H). **$^{31}$P{$^{1}$H} NMR (121 MHz, $CDCl_3$):** δ 75.5 ppm.

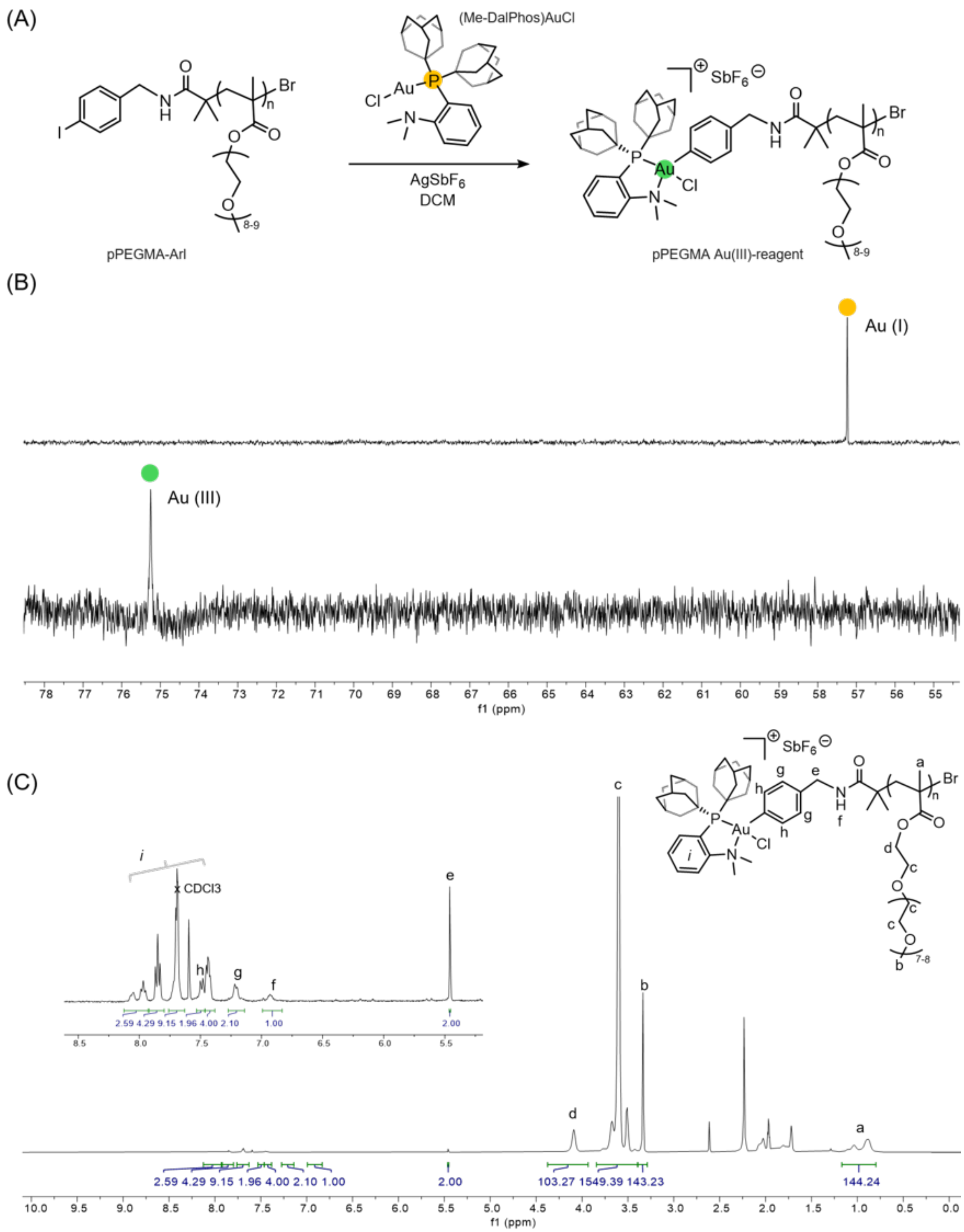


**Figure S14.** (A) Synthetic scheme for the preparation of pPEGMA Au(III)-reagent from pPEGMA-ArI (initiated by **2'**). (B) Comparison of $^{31}$P NMR spectra showing the conversion of Au(I) precursor to the corresponding Au(III) complex. (C) $^{1}$H NMR spectrum ($CDCl_3$) of the pPEGMA Au(III)-reagent with peak assignments before bioconjugation (inset: magnified aromatic and amide region showing unreacted MeDalPhos-Au(I)Cl.)

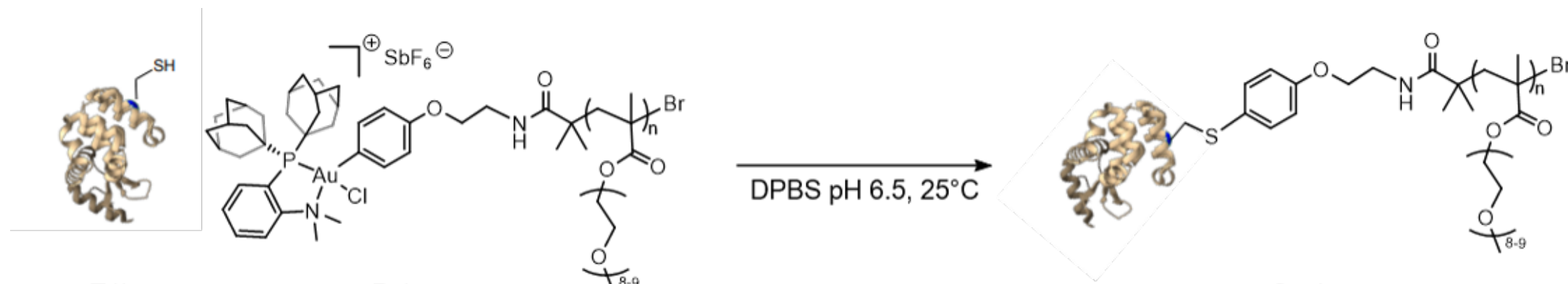


T4L Polymer Conjugate

**Coomassie**

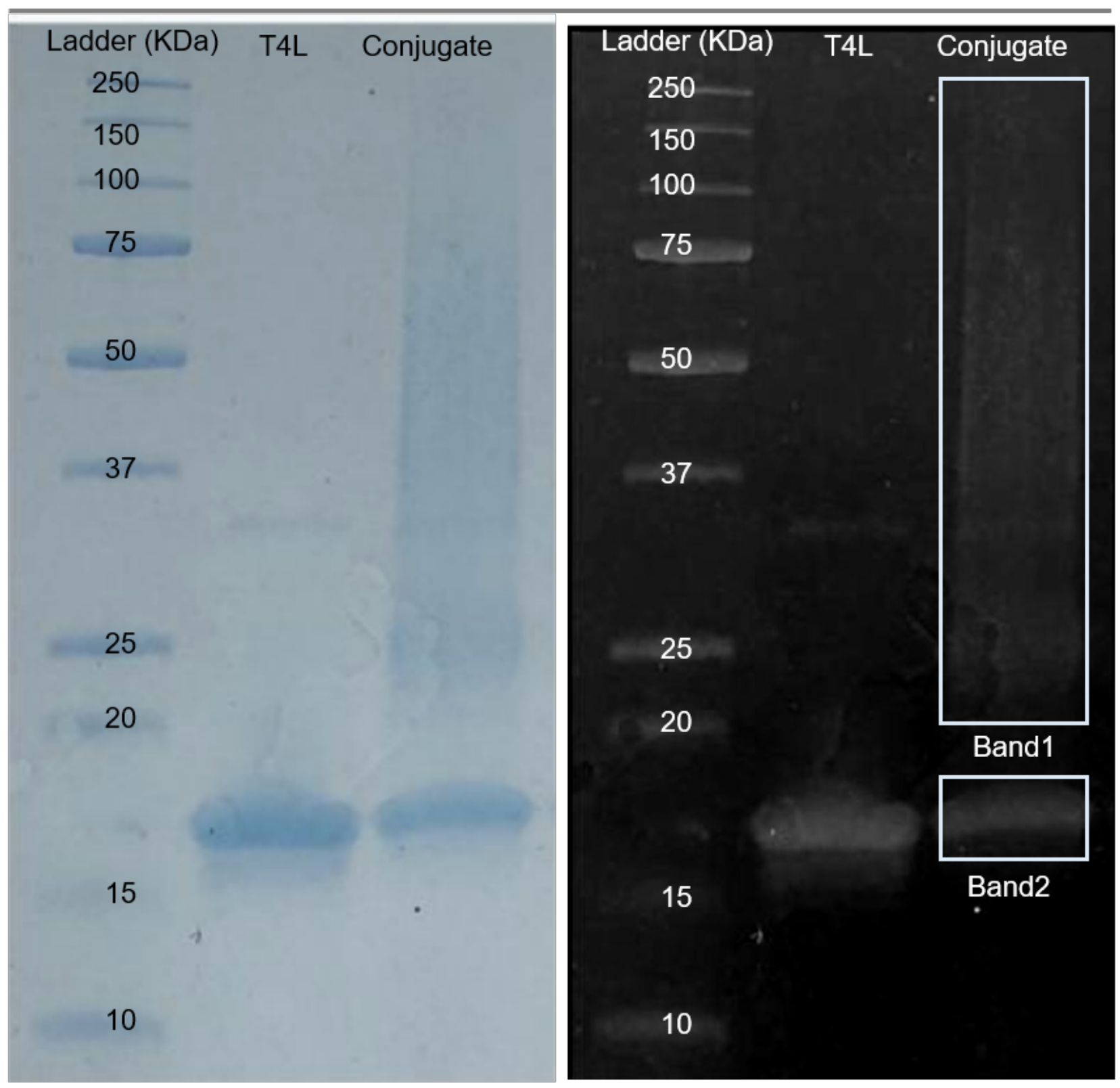


| Band | Integrated density |
|---|---|
| 1 (T4L-pPEGMA) | 1,384,932 |
| 2 (Unreacted T4L) | 111,012 |

$$\text{Conversion (\%)} : \frac{\text{Integrated density}_{(\text{T4L-pPEGMA})}}{\text{Integrated density}_{(\text{T4L-pPEGMA})} + \text{Integrated density}_{(\text{Unreacted T4L})}} \text{ X } 100$$

$$: \frac{1{,}384{,}932}{(1{,}384{,}932 + 111{,}012)} \text{ X } 100 = 92.6\ \%$$

**Figure S15.** Gel electrophoresis (SDS-PAGE) from **Figure 3b (left)** and Image J analysis of the T4L-pPEGMA conjugate. Below are the integrated densities from the bands. Conversion was calculated based on integrated density in the conjugate SDS Page. Specifically, the T4-pPEGMA signal was compared to the unreacted T4 in the same lane. From this, the conversion was 92.6%.

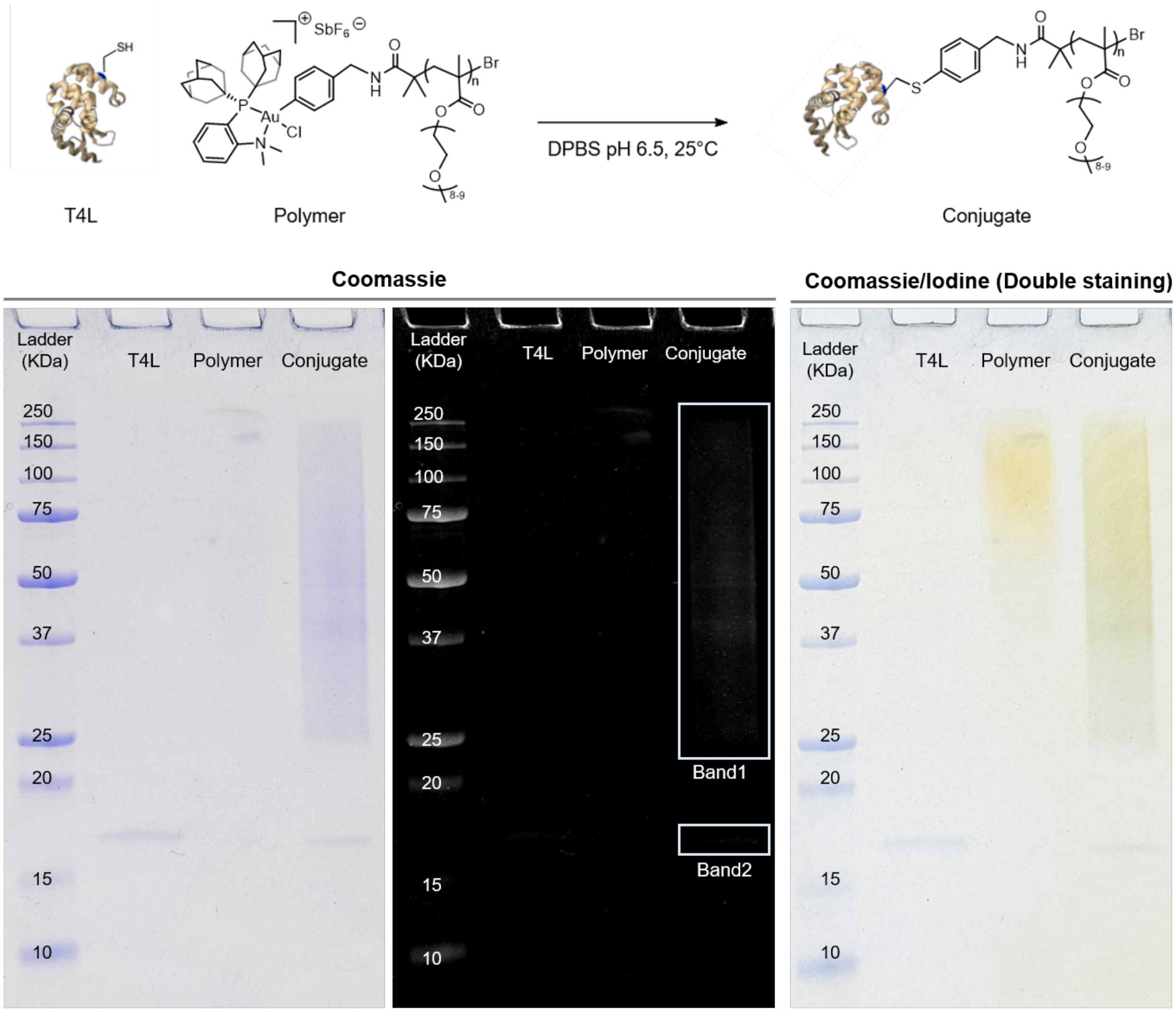


| Band | Integrated density |
|---|---|
| 1 (T4L-pPEGMA) | 58,542 |
| 2 (Unreacted T4L) | 4,791 |

$$\text{Conversion: } \frac{58{,}542}{(58{,}542 + 4{,}791)} \times 100 = 92.4\ \%$$

**Figure S16.** SDS-PAGE and ImageJ analysis of the T4L-pPEGMA conjugate for the versatility test (pPEGMA was synthesized using initiator **2'**). Coomassie/iodine double-stained gel demonstrating successful bioconjugation, where the iodine stain selectively visualizes PEG polymers as a distinct yellow band in the polymer lane. Integrated density profiles extracted to determine bioconjugation efficiency; the T4L-pPEGMA conjugate signal was compared to the unreacted T4L within the same lane, yielding a conversion efficiency of 92.4% (consistent with **Figure S15**). Note that the background signals were uniformly subtracted to ensure accurate integrated density quantification.

**Solid-Phase Peptide Synthesis**

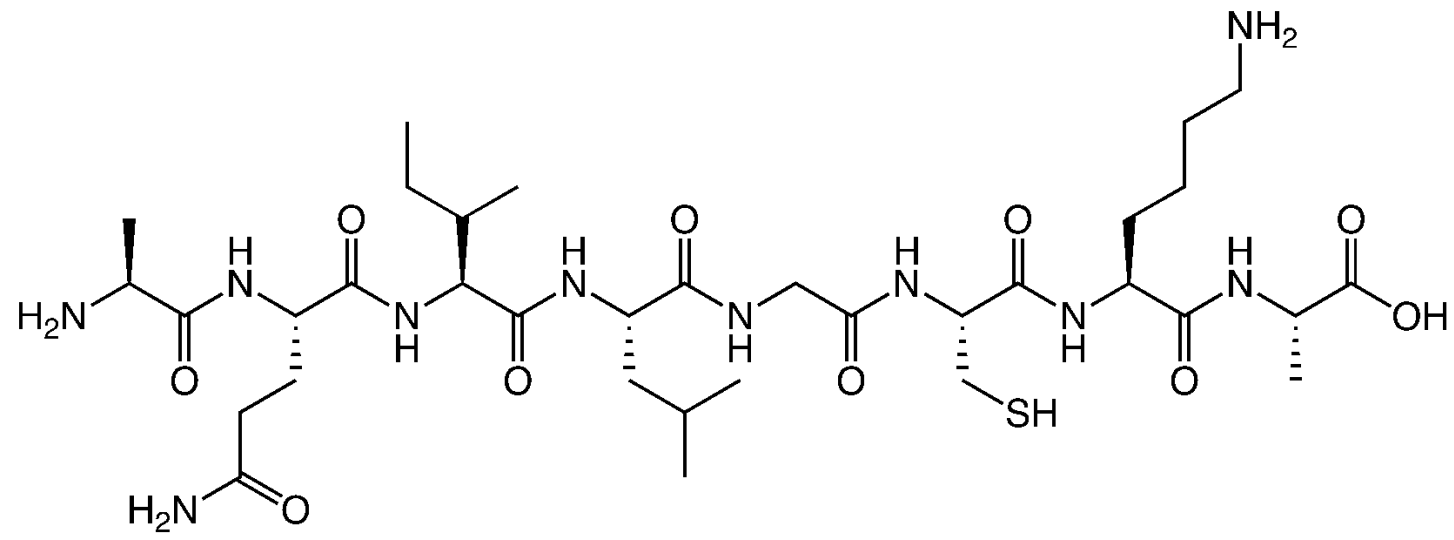

The peptide was synthesized via standard Fmoc solid-phase peptide synthesis conditions. The peptide was prepared using 1.000 g of 2-chlorotrityl chloride resin (0.73 mmol/g ChemImpex). The resin was swelled in Dichloromethane (DCM) for 60 min prior to any modifications. The initial alanine coupling was carried out using two equivalents of Fmoc-alanine-OH and 6 equivalents of N,N-Diisopropylethylamine (DIPEA). This was done in 12 mL of 1:1 DCM and N-Methylpyrrolidone (NMP) (v/v) mixture and shaken for 90 min. Each amino acid residue thereafter was loaded using a 3:3:6 equivalent ratio of Fmoc-amino acid: HATU: DIPEA in NMP for 30 min. The couplings were followed by deprotection in 20% 4-methylpiperidine in DMF (v/v, ca. 8 mL) for 20 min. For the coupling of Fmoc-cysteine(Trt)-OH, 6 equivalents of 2,4,6-trimethylpyridine were used to prevent racemization. After the couplings were complete, the resin was dried under vacuum for 30 min and then swelled in DCM for 40 min. The peptide was cleaved from the resin while simultaneously removing protecting groups by using a 20% Trifluoroacetic acid (TFA) and 5% TIPS in DCM (v/v) cleavage cocktail. Approximately 10 mL of the cleavage cocktail was added to the resin/peptide and mixed for two min. The solution was filtered and the solvent removed under vacuum. This process was repeated three additional times. After removal of TFA and DCM under vacuum, the concentrate was precipitated into chilled diethyl ether (45 mL, ca. -20 °C) to afford the crude product as a white solid. The resulting crude product was then purified *via* preparative HPLC (10-80% acetonitrile (MeCN) gradient against water, both with a 0.1% TFA additive) to afford the product as a white solid.

**HRMS (ESI/QTOF):** $[M+H]^+$ calculated $C_{34}H_{62}N_{10}O_{10}S^+$ 803.4443, observed 803.4424.

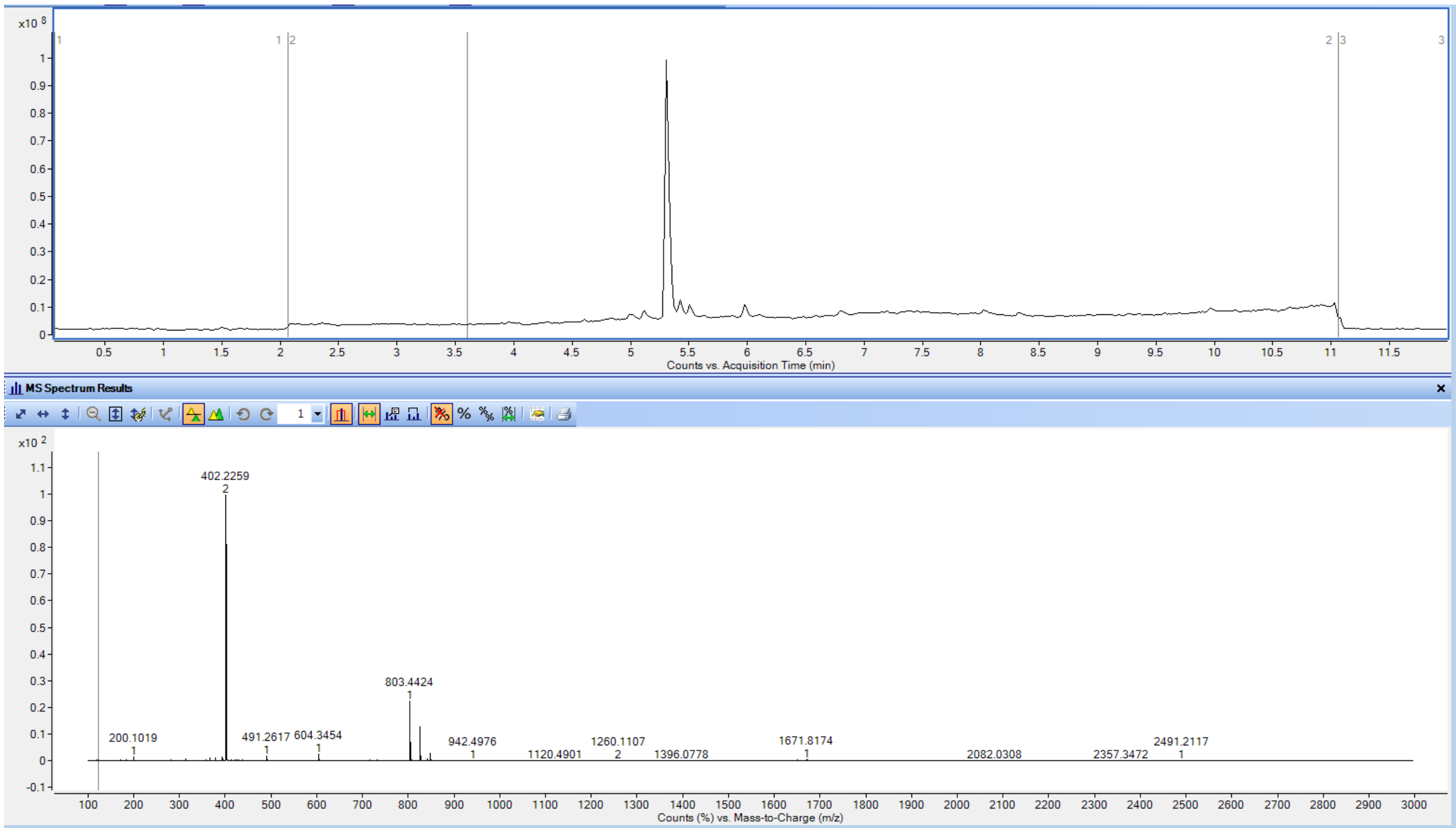


**Figure S17.** LCMS of purified synthetic peptide. Top: TIC, bottom: intact mass of peptide with masses for z = 1 and z = 2.

## T4 Lysozyme Expression

T4 Lysozyme (V131C) protein expression and purification was adapted from literature procedure.[2] T4 Lysozyme (V131C) Sequence (Calculated Mass: 18605.36 Da): MNIFEMLRIDEGLRLKIYKDTEGYYTIGIGHLLTKSPSLNAAKSELDKAIGRNTNGVITKD EAEKLFNQDVDAAVRGILRNAKLKPVYDSLDAVRRAALINMVFQMGETGVAGFTNSLR MLQQKRWDEAACNLAKSRWYNQTPNRAKRVITTFRTGTWDAYKNL. *E. coli* host BL21(DE3) (Invitrogen) was used to express V131C T4 lysozyme using an ampicillin resistant expression vector obtained from Prof. Wayne Hubbell (UCLA). Plasmid induced *E. coli* glycerol stocks were cultured in LB Broth (Miller) at 37 °C with shaking overnight. The culture was grown at 37 °C with 250 rpm shaking for about 6 hours before the OD600 reached ~0.75 and the culture was induced with 1 mM Isopropyl β-D-1-thiogalactopyranoside (IPTG). The culture was continued to shake at 37 °C at 250 rpm for approximately 2 hours. The cultures were harvested by centrifugation at 4000 rpm for 15 min to yield a cell pellet. The pellet was resuspended in 20 ml lysis buffer (25 mM tris(hydroxymethyl)aminomethane (Tris), 25 mM (3-(N-morpholino)propanesulfonic acid) (MOPS), 0.2 mM Ethylenediaminetetraacetic acid (EDTA), pH 7.6) by stirring vigorously. Spontaneous bacterial lysis occurred during resuspension as evident by increased viscosity of the suspension due to DNA release. Bacterial lysis was likely due to proteolytic activity of the lysozyme present in the bacteria. One subsequent freeze-thaw cycle ensured complete lysis of the bacteria. Benzoase (0.2μL/mL) was then added to the solution and incubated at 25 °C for 15 min. Bacterial debris was then separated by centrifugation at 17,000 rpm for 25 min at 4 °C. The supernatant was loaded onto a 5 mL gravity SP Sepharose Fast Flow column (Cytiva) and washed with 200 mL lysis buffer. The column was then washed with two column volumes of lysis buffer with increasing NaCl content (gradient from 0M to 0.5 M in 0.1 M steps) to elute the desired protein. SDS-PAGE was run on all fractions and under reducing conditions with Coomassie Blue staining. Pure fractions were combined. and the solvent exchanged into storage buffer (20 mM PBS, pH 6.5) and concentrated to ~15 mL using a using Amicon 3K Ultra-15 Centrifugal Filter (Millipore). Protein was further purified by preparative SEC FPLC using an isocratic method in 20 mM PBS, pH 6.5. (The FPLC purification as shown in Figure S15) Pure fractions were concentrated and stored in storage buffer as described above. The purified protein was analyzed by LC-MS and SDS-PAGE (Figure S16) confirming sample purity and molecular weight. Concentration was determined by A280 (Extinction coefficient = 25,440 $M^{-1}$ $cm^{-1}$) The protein sample was diluted with storage buffer to 76 μM and aliquots were flash frozen and stored in a –80 °C freezer.

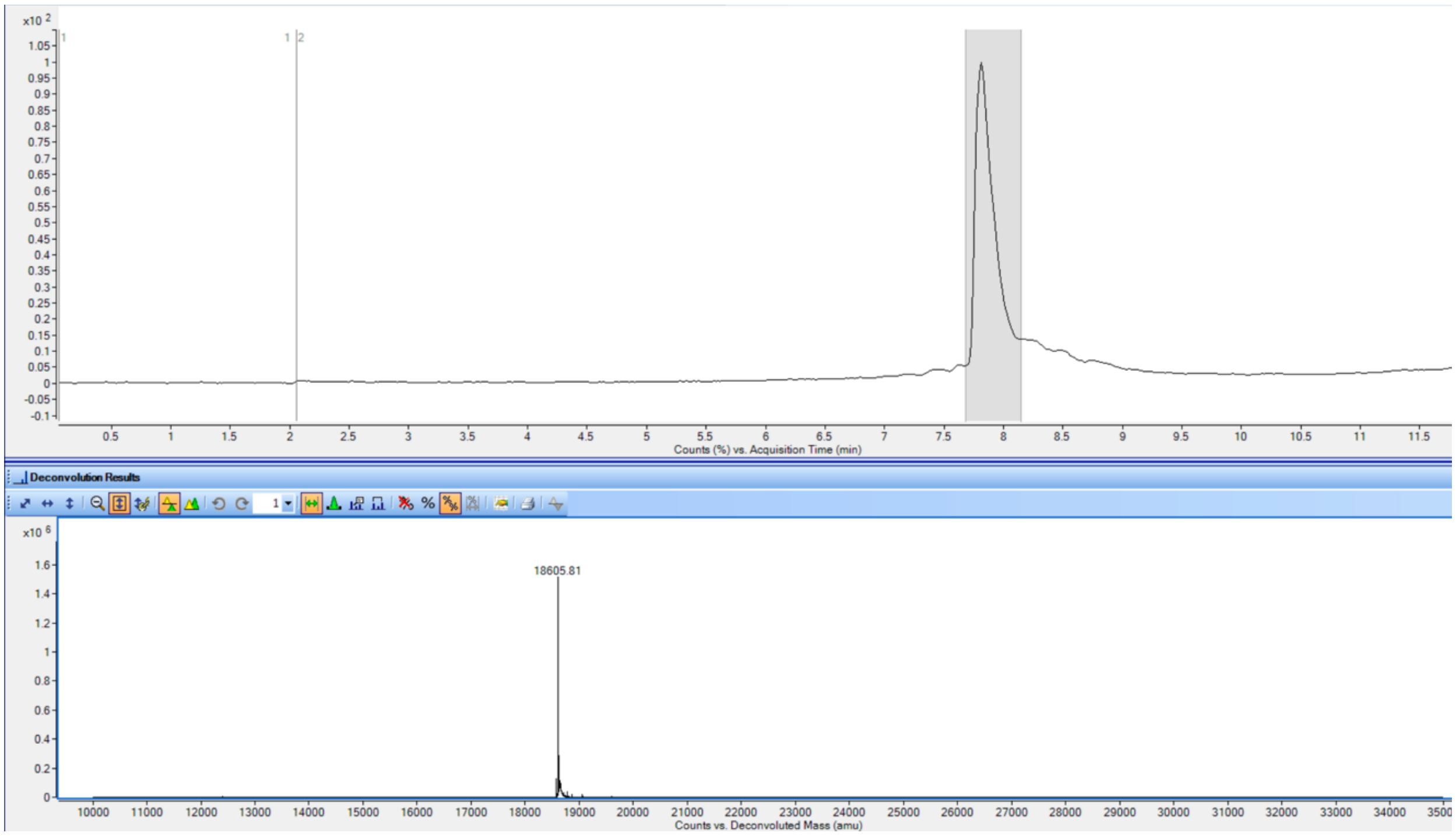


**Figure S18.** LCMS of purified protein T4 Lysozyme (V131C). Top: TIC, bottom: mass of T4L.

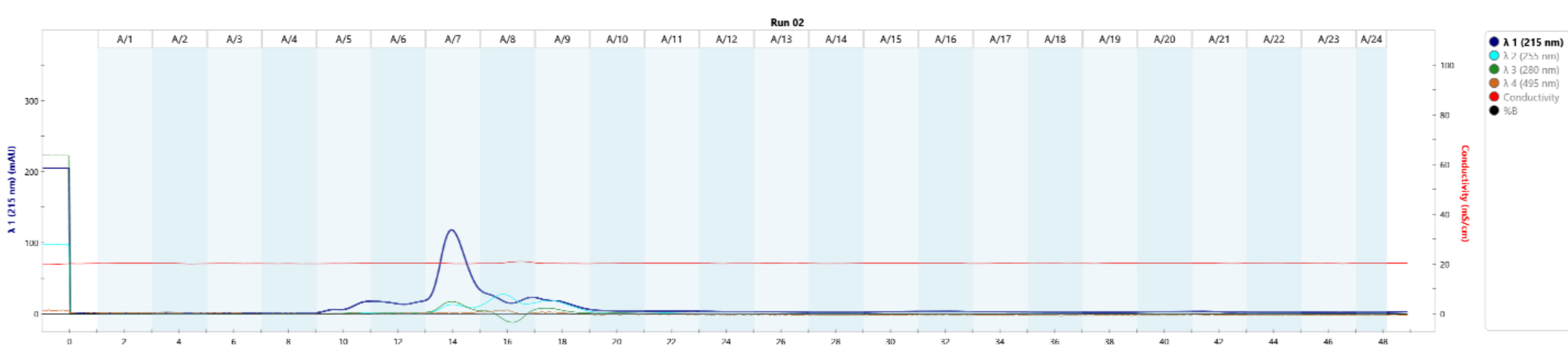


**Figure S19.** Purification of T4 Lysozyme (V131C) protein via FPLC (SEC column, eluent: 20 mM PBS, pH 6.5).

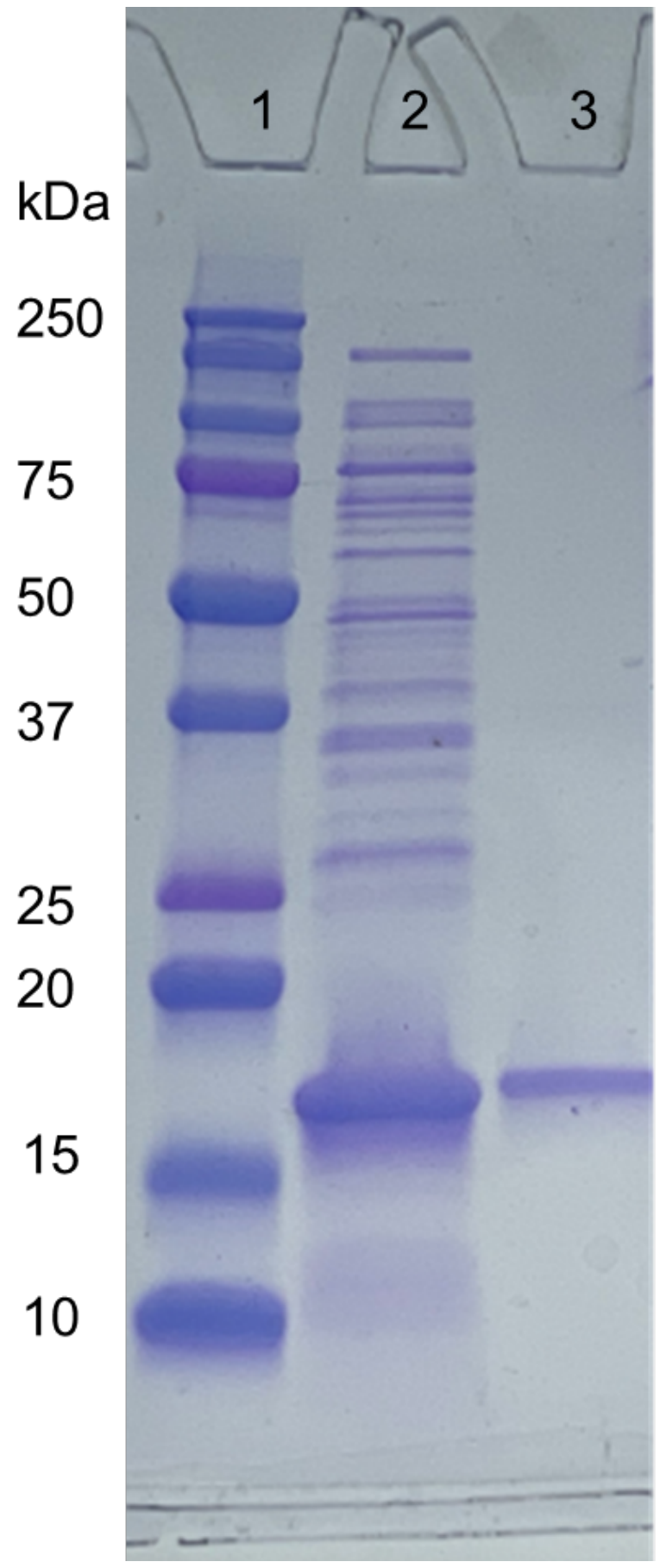


**Figure S20.** Gel electrophoresis (SDS-PAGE) analysis of the purified T4 Lysozyme (V131C) protein.
Lane 1: Ladder; Lane 2: Crude T4L; Lane 3: purified T4L.

## Au(III)-pPEGMA T4 Lysozyme Conjugation

To 50 µL of a solution of 76 µM T4 lysozyme in DPBS pH 6.5, was added 5 µL of a 3 mM stock solution of TCEP•HCl (4 equivs.) in MilliQ $H_2O$. This solution was allowed to sit at 25 °C for one hour to reduce any intraprotein disulfide bonds formed during storage. Then, 5 µL of a 1 mM stock solution of Au(III)-pPEGMA reagent (1.3 equivalents) in MilliQ $H_2O$ was added, gently flicked to mix and allowed to react for five min. After five min, the reaction was stopped by dilution in Lameilli loading buffer (5% β-mercaptoethanol) and run on SDS-PAGE for analysis.

## Peptide Macroinitiator Synthesis

To 500 µL of a solution of 1 mM peptide in 20 mM Tris, 150 mM NaCl, pH 7.5, 500 µL of a 1 mM stock solution of Au(III)-initiator (1.2 equivalent) in MeCN was added, vortexed to mix, and allowed to react for five min. The resulting crude peptide macroinitiator was then purified via preparative HPLC (10-80% acetonitrile (MeCN) gradient against water, with a 0.1% TFA additive, column: Luna 5 µm C18 100 Å column, flow rate: 20 mL $min^{-1}$) to yield pure macroinitiator (yield 67%). The macroinitiator was characterized by LC-MS **(see Figure 5a).**

## T4L Macroinitiator Synthesis

To 700 µL of a solution of 76 µM T4L in 20 mM Tris, 150 mM NaCl, pH 7.5 was added 70 µL of a 3 mM stock solution of TCEP•HCl (4 equivs.) in MilliQ $H_2O$. This solution was allowed to sit at 25 °C for one hour. Then, 70 µL of a 1 mM stock solution of Au(III)-initiator (1.3 equivalents) in MeCN was added, gently flicked to mix and allowed to react for five min. This macroinitiator was purified using FPLC SEC chromatography (Eluent: 20 mM Tris buffer, 150 mM NaCl, pH 7.5, room temperature, Column: ENrich SEC 650 10 x 300 Column, flow rate: 1.0 mL $min^{-1}$). The macroinitiator was characterized by SDS Gel electrophoresis (see **Figure 6e, lane 3**) and LC-MS **(see Figure 5b)**.

**Peptide-Polymer Conjugates via Grafting From SET-LRP**

This procedure was adapted from literature procedure.[3] $Me_6TREN$ (0.48 µL, 2.4 µmol, 1.3 equiv.) was dissolved in 250 µl $H_2O$ and sparged with argon for 5 min. Then, this solution was added to a vial of Cu(I)Br (0.34 mg, 2.4 µmol, 1.3 equiv.) and this mixture was sparged for an additional 15 min at 0 °C while stirring. A purple Cu(0) catalyst precipitated from the blue solution. To this solution was added a solution of DMAEMA monomer (29 mg, 0.18 mmol, 100 equiv.) and peptide macroinitiator (2 mg, 1.8 µmol, 1 equiv.) dissolved in 500 µl $H_2O$ which was also previously sparged with argon for 15 min at 0 °C. The reaction was stirred for 20 min at 0 °C and became thick. The water was removed by lyophilization and the polymer was purified by precipitation in diethyl ether.

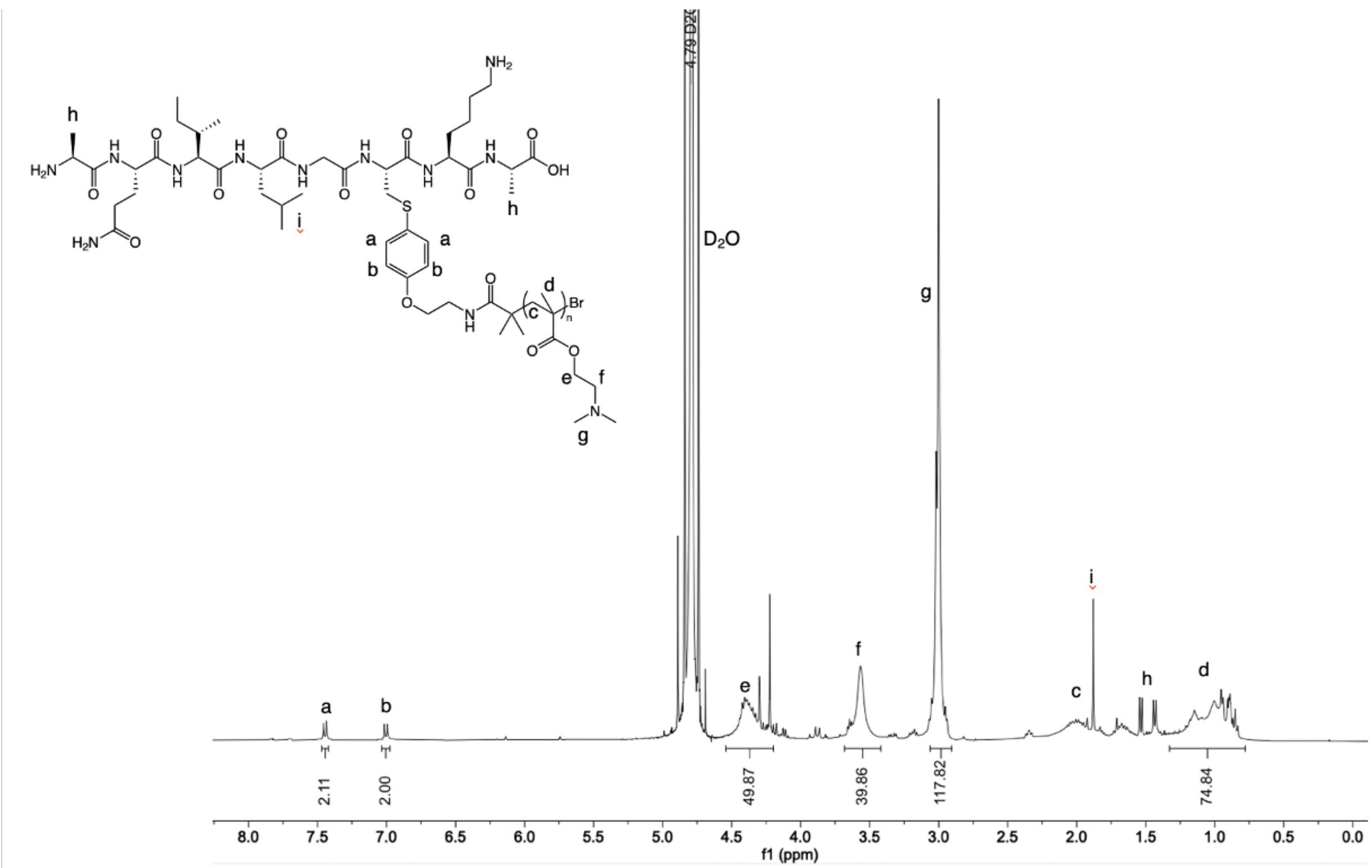


**Figure S21.** $^1H$ NMR spectrum of peptide-pDMAEMA in $D_2O$ at 25 °C.

### T4L-Polymer Conjugate *via* Grafting From SET-LRP

This procedure was adapted from literature procedure.[4] The ATRP catalyst stock solution composed of $CuBr_2$ (10 mM) and tris(2-pyridylmethyl)amine (TPMA) (100 mM) in PBS (10 mM) was prepared with 10 % DMF (v/v) as a co-solvent. 76 µL of the ATRP catalyst stock solution was added to the mixture of 1000 µl of a 76 µM solution of T4L-Br (0.076 µmol, 1 equiv.), 144.4 µl of a 10 mM solution of sacrificial initiator 2 (1.444 µmol, 19 equiv.), and DMAEMA (28 µL, 152 µmol, 2000 equiv.). The reaction mixture was put into a Schlenk tube, sealed with a rubber septum, and deoxygenated by sparging with nitrogen for 20 min at 0 °C. 76 µl of a 100 mM ascorbic acid (AscA) solution (7.6 µmol, 100 equiv.) was carefully sparged with argon for 10 min. This solution was then added to the reaction mixture to begin the reaction with magnetic stirring. After 4 h, the polymerization was quenched by bubbling with air. Additionally, the T4L-pDMAEMA bioconjugate (1 mg/mL) was incubated with proteinase K (0.5 mg/mL) in digestion buffer (50 mM Tris·HCl, 2 mM $CaCl_2$, pH 7.4) at 40 °C for 48 h before analysis for the estimation of molecular weight of pDMAEMA.

**SEC** (DMF) **:** $M_n^{SEC}$ = 62.2 kDa, $M_w^{SEC}$ = 111.4 kDa, $Đ$ = 1.79.

**SEC** (Aqueous) **:** $M_n^{SEC}$ = 90.6 kDa, $M_w^{SEC}$ = 155.9 kDa, $Đ$ = 1.72.

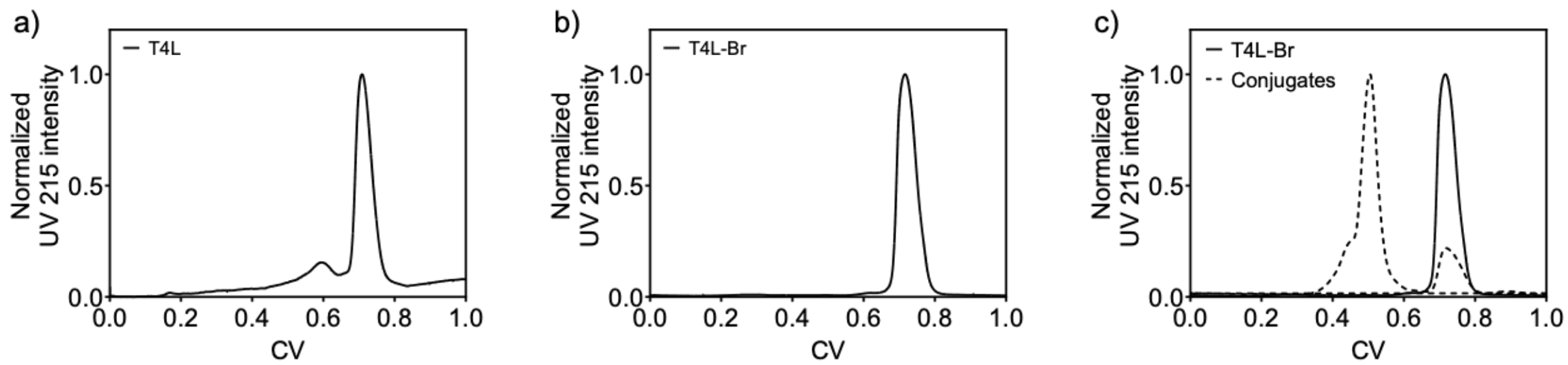


**Figure S22.** FPLC SEC confirms synthesis of pDMAEMA from protein T4L-Br macroinitiator. SEC traces of a) T4L, b) T4L-Br, c) T4L-Br and T4L-pDMAEMA.

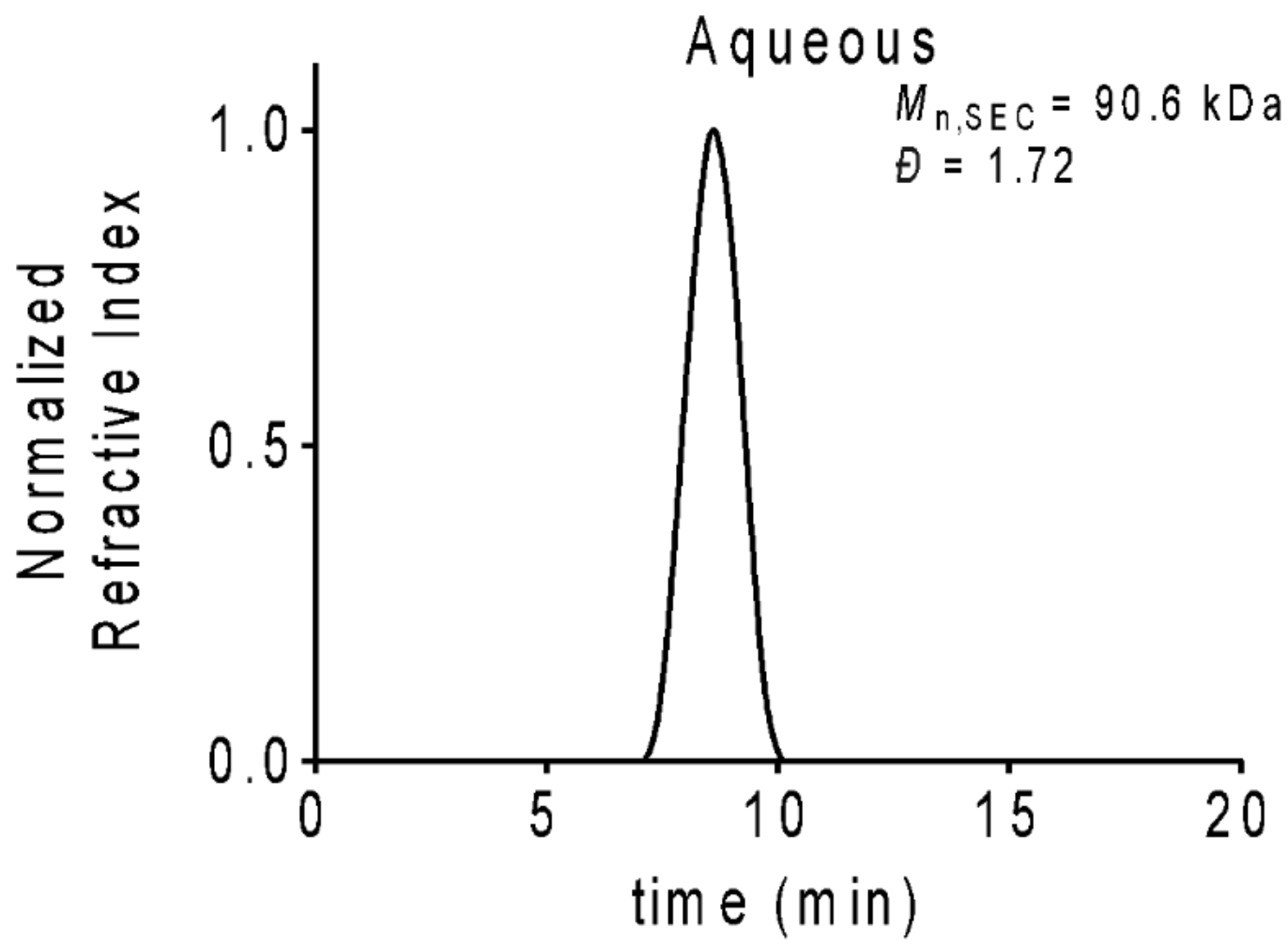


**Figure S23**. SEC confirms synthesis of pDMAEMA (digested form T4L-pDMAEMA conjugates using proteinase K) acquired from aqueous SEC compared to Shodex STANDARD P-82 standards using PBS (20 mM phosphate, pH 5.0 with 500 mM NaCl) as eluent.

## References


1) Wang, X.-S.; Lascelles, S. F.; Jackson, R. A.; Armes, S. P. Facile Synthesis of Well-Defined Water-Soluble Polymers via Atom Transfer Radical Polymerization in Aqueous Media at Ambient Temperature. *Chem. Commun.* **1999**, No. 18, 1817–1818. https://doi.org/10.1039/A904691G.
2) Mchaourab, H. S.; Lietzow, M. A.; Hideg, K.; Hubbell, W. L. Motion of Spin-Labeled Side Chains in T4 Lysozyme. Correlation with Protein Structure and Dynamics. *Biochemistry* **1996**, *35* (24), 7692–7704. https://doi.org/10.1021/bi960482k.
3) Zhang, Q.; Li, M.; Zhu, C.; Nurumbetov, G.; Li, Z.; Wilson, P.; Kempe, K.; Haddleton, D. M. Well-Defined Protein/Peptide–Polymer Conjugates by Aqueous Cu-LRP: Synthesis and Controlled Self-Assembly. *J. Am. Chem. Soc.* **2015**, *137* (29), 9344–9353. https://doi.org/10.1021/jacs.5b04139.
4) Mansfield, K.M.; Maynard, H.D. Site-specific insulin-trehalose glycopolymer conjugate by grafting from strategy improves bioactivity. *ACS Macro Lett*. **2018**, 7(3), pp.324-329. https://doi.org/10.1021/acsmacrolett.7b00974.
